# The engagement equation: a model for understanding what drives voluntary physician engagement with data-driven clinical performance feedback

**DOI:** 10.1186/s43058-025-00819-5

**Published:** 2025-12-11

**Authors:** Laura Desveaux, Ruoxi Wang, Simona C. Minotti, Benjamin Brown, Alexandra Harris, Amol Verma, Geneviève Rouleau, Mina Tadrous, Braeden Terpou, Noah M. Ivers

**Affiliations:** 1 Institute for Better Health, Trillium Health Partners, 2085 Hurontario St, Mississauga, ON L5A 4G1 Canada; 2 Institute for Health Policy, Management, and Evaluation, University of Toronto, Toronto, ON Canada; 3 Women’s College Hospital Research Institute, Women’s College Hospital, Toronto, ON Canada; 4 Division of Biostatistics, Dalla Lana School of Public Health, University of Toronto, Toronto, ON Canada; 5 School of Health Sciences, Division of Population Health, Health Services Research and Primary Care, University of Manchester, Williamson Building, Oxford Road, Manchester, M13 9PL UK; 6 Li Ka Shing Knowledge Institute, Unity Health, Toronto, ON Canada; 7 Department of Medicine, Temerty Faculty of Medicine, University of Toronto, Toronto, ON Canada; 8 Nursing Department, Université du Québec en Outaouais, Gatineau, Québec Canada; 9 Institut du Savoir Montfort, Ottawa, ON Canada; 10 Leslie Dan Faculty of Pharmacy, University of Toronto, Toronto, ON Canada; 11 Department of Family and Community Medicine, University of Toronto, Toronto, ON Canada

## Abstract

**Background:**

Clinical performance feedback (CPF) is widely used to support physician development and improve care. Yet, its impact remains limited by low voluntary engagement. This study sought to: (1) develop a theory-informed, report-agnostic model outlining the key beliefs that shape physician engagement with CPF; (2) explore patterns of feedback orientation across physicians; and (3) understand how individual perceptions influence engagement with CPF.

**Methods:**

We used a cross-sectional, multi-method approach combining a survey and qualitative interviews with primary care physicians in Ontario, Canada. We validated a conceptual model using path analysis, explored heterogeneity in feedback orientation using latent profile analysis, and qualitatively examined how perceptions of CPF influenced engagement.

**Results:**

Survey results (*n *= 206) supported a model in which engagement with CPF is shaped by five recipient characteristics: perceived need for change (change discrepancy), perceived value of CPF, confidence to act on feedback (feedback self-efficacy), belief that feedback is useful (feedback utility), and sense of responsibility to act (feedback accountability). Perceived utility mediated the effects of self-efficacy and value on accountability, and perceived need for change influenced value. Latent profile analysis identified three groups: physicians with high and balanced feedback orientation (*n *= 32), moderate and balanced (*n *= 143), and low feedback orientation with low self-efficacy (*n *= 31). Interview findings (*n *= 9) revealed two mindsets: physicians who saw value in CPF despite its limitations (engagers), and those who dismissed its relevance (non-engagers). These mindsets aligned with differences in value, utility, and accountability scores from the survey.

**Conclusions:**

Engagement with CPF is not one-size-fits-all. Physicians differ in how they appraise and act on feedback based on their beliefs about its relevance, usefulness, and their ability to act. CPF initiatives should explicitly link feedback to improved patient outcomes, focus on future actions, and provide clear, actionable guidance. Designing CPF that accounts for recipient heterogeneity is essential to realizing its full potential as an improvement strategy.

**Supplementary Information:**

The online version contains supplementary material available at 10.1186/s43058-025-00819-5.

Contributions to the literature

- This study advances the science by specifying recipient-level determinants—change discrepancy, value, self-efficacy, utility, and accountability—that shape engagement with clinical performance feedback (CPF), a core implementation strategy.
- By specifying high-value determinants that drive engagement, these results reframe engagement not as a passive outcome, but as an active, theory-informed implementation target, shifting focus from design features alone to how CPF is appraised by recipients.
- These findings demonstrate that engagement is shaped by identifiable and modifiable beliefs, offering a practical model to tailor CPF initiatives to recipient profiles and increase the effectiveness of feedback interventions.

## Background

Clinical performance feedback (CPF), also called audit and feedback, is a widely-used implementation strategy where a clinician’s performance is measured, compared to professional standards or target, and fed back to them to improve care outcomes at scale [1]. Ivers et al. [2] highlight a wide range in effect sizes for continuous outcomes, with an interquartile range of 1.3% to 26.1%, demonstrating that CPF can have a significant impact when implemented effectively. Unfortunately, this impact often remains untapped, due in part to low levels of clinician engagement [3, 4]. Engagement in this context is defined as the upstream, cognitive interaction with one’s personalized feedback, where participants voluntarily access and actively review the data, paying attention to its content. It requires that participants have access to CPF, and is a necessary step before any decision to use CPF to identify gaps or change practice. To date, CPF research has primarily focused on whether CPF works on average, however limited attention to engagement leaves opportunities for better patient outcomes unrealized.

Clinical Performance Feedback Intervention Theory (CP-FIT) outlines the necessary pathway for improvements in patient care, identifying three sets of variables that influence the feedback cycle: feedback variables, recipient characteristics, and the broader practice context [5]. While it is known that characteristics of CPF – its design, content, and delivery – influence its effectiveness [2, 6, 7], far less is known about how individual characteristics affect whether a recipient engages with it (or not) in the first place. Brown et al. [5] describe that positive attitudes with respect to feedback (i.e., views on the potential benefits) increased the likelihood of engagement. However, the full range of important recipient characteristics that might influence engagement with CPF and how they interact with one another has not yet been systematically explored. Simply put, the pathway(s) for engagement in CPF remain unclear, limiting our ability to target them with evidence-based approaches.

In addition to suboptimal engagement, there is heterogeneity in responsiveness to CPF across recipients [8, 9]. In the broader feedback literature, the perception of feedback delivery (e.g., whether the feedback is delivered in a non-judgemental manner) has a positive association with reactions to feedback among older recipients, while feedback quality (e.g., whether the feedback is relevant, specific, consistent, and detailed) has a positive association with reactions among younger recipients [10]. These insights highlight that recipient subgroups process feedback differently [10, 11], but how perceptions of feedback affect engagement remains unclear. Preliminary evidence suggests that differences in perceived need for change, feedback attitudes (often called feedback orientation), and perceived value influence engagement among primary care physicians [12]. However, it is necessary to more fully understand which variables influence recipient perceptions of CPF and how they might interact to influence (or undermine) its effectiveness as an implementation strategy.

The Necessity-Concerns Framework (NCF) [13] offers a useful lens to explore these dynamics as it emphasizes the balance individuals consider between the perceived benefits (necessity) and drawbacks (concerns) of an action. While the NCF provides a structured approach to understanding how patients approach medication decisions, the underlying model of cognitive appraisal may extend to CPF. Physicians, like patients, evaluate the benefits and risks of actions when deciding whether or not to engage with CPF, including how relevant it is to improving patient outcomes [14], the effort required to act on it [15], and potential consequences (e.g., criticism or increased workload) [16]. For example, if a physician sees data as a chance to improve patient care (necessity) but worries it might reveal a weakness or lead to extra work (concerns), the tension between these perceptions could determine whether they engage with the data. A deeper examination of how physicians cognitively appraise feedback—such as its relevance, specificity, and utility—might drive or deter their responsiveness to such performance data.

Over the last decade, we have conducted a series of studies qualitatively exploring reactions to and experiences with CPF [12, 17–19]. Together, this work suggested that the beliefs about *feedback accountability* influenced engagement with CPF with at least two antecedents: *feedback self-efficacy* and perceived *feedback value.* These studies highlight the important influence physician characteristics have on the effectiveness of CPF and suggest that ‘engagement’ is the endpoint of a cognitive appraisal process following which physician recipients would decide whether to actively engage with or use CPF to identify performance gaps and make changes in their practice. To systematically understand the physician characteristics that influence engagement with CPF, this work sought to (1) develop an applied, report-agnostic model to specify the constructs influencing engagement with CPF; (2) explore recipient heterogeneity in feedback orientation; and (3) qualitatively explore heterogeneity in perceptions of CPF and their impact on engagement. Given their influence on CPF, we build on prior work by clarifying recipient characteristics that influence engagement and how they interact. Insights from this work will support the identification of evidence-informed strategies to increase upstream engagement with CPF to realize its potential impact on patient outcomes.

### Model development

We began our review of the literature with CP-FIT [5] – a comprehensive theory of CPF in a healthcare context - which acknowledges the role of attitudes towards feedback as an upstream influence but does not specify whether these characteristics interact or how they influence perceptions of CPF. Next, we sought out a feedback-specific theory that focused on recipient beliefs. Linderbaum and Levy’s [17] Feedback Orientation Scale (FOS) centres around attitudes towards feedback, highlighting sub-domains of feedback self-efficacy, feedback utility, feedback accountability, and social awareness. While these sub-domains provide a strong foundation for understanding recipient beliefs that might influence engagement with CPF, two key knowledge gaps remained: the relationships between FOS sub-domains (given that most studies treat feedback orientation as a single concept [18, 19]) and the influence of perceived value – a known antecedent to behavioural intention [20, 21]. We then searched the literature related to FOS domains to generate hypotheses for testing.

### Feedback self-efficacy and value as antecedents of feedback accountability

Linderbaum and Levy [17] defined *feedback accountability* as an individual’s perceived sense of responsibility for acting on feedback, making it a precursor to intention to act on specific feedback information as well as an outstanding driver of the behavioral response itself. Studies that have investigated the subdomains of feedback orientation conceptualized *feedback accountability* as a *commitment to action*, which is a more behaviour or action-oriented feature than the subdomains of *feedback utility* and *feedback self-efficacy* [22, 23].

*Feedback self-efficacy* refers to individuals’ confidence in their ability to act on feedback [17]. The broader concept of *self-efficacy* has been widely investigated in various theories including Social Cognitive Theory [24], the Theory of Planned Behavior [21], and Social Cognitive Career Theory [25], with documented positive impact on both intention and behaviour. It has been conceptualized as an individual’s cognitive appraisal of their control over achievement and demonstrated as one of the two most important individual antecedents of behaviour from the lens of Control-Value Theory [26, 27]. In the feedback orientation literature, Yang and Yang [22] linked *feedback self-efficacy* to *feedback accountability* and action (behaviour): individuals who have higher confidence in their ability to deal with feedback are more likely to feel responsible for acting on feedback, and accordingly, act on feedback more proactively.

In addition to *self-efficacy*, Control-Value Theory [20] suggests *value* as the second individual antecedent of action. *Value* refers to individuals’ cognitive appraisal of a given action (in the case, the action of interacting with CPF reports) in terms of its personal relevance (i.e., whether the outcome of action matters to individuals). *Value* has been demonstrated as an important driver of action in Expectancy-Value Theory [28], the Theory of Planned Behavior [21], and Health Belief Model [29]. *Self-efficacy* and *value* have been shown to have independent influence on action (i.e., individuals can be best motivated if they have confidence in their ability to act and the corresponding outcome is important to them), highlighting the importance of investigating these two factors simultaneously [26].

### Mediating effect of feedback utility

Feedback *utility* refers to an individual’s perceived usefulness of feedback- a context-specific judgement that a CPF report will help them improve [17]. Utility has been widely investigated in the information system and marketing fields where it operates as a property of a product or system. In the case of CPF, *utility* is the belief that CPF enables learning and performance improvement. This is distinct from the construct of *value* that manifests as an individual/consumer appraisal of importance or relevance [30]. *Value* answers the question “Does this matter?” while *utility* asks “Will this help me improve?”. Consider two physicians receiving the same report. Dr. A views CPF as valuable (it matters for patient care) but is less convinced it can help them improve, so they skim the report at best and disregard it at worst. Dr. B views CPF as valuable and useful, reviewing it closely and identifying a change in response. Thus, value supports attention, whereas usefulness supports the reflection necessary to identify an action. Prior studies show that *utility* mediates effects of *self-efficacy* and *value* on intention (Alalwan et al. [31], Wang et al. [32], and Youn and Lee [33]). Alam et al. [34] and Han and Nam [35] further demonstrated the independent positive effects of *self-efficacy* and *value* on *utility* as well as the positive effect of *utility* on intention. In the feedback orientation literature, Frondozo and Yang [23] validated the mediating role of *utility*, explaining the effect of *self-efficacy* on *feedback accountability*: individuals who have higher confidence in their ability to deal with feedback tend to perceive feedback as more useful, and as a result, feel higher responsibility for acting on feedback. However, whether the *value* influences *utility* in the context of CPF, and whether *utility* influences *accountability* warrants further investigation.

These literature supported the following hypotheses:

- *Hypothesis 1. Feedback self-efficacy has a positive relationship with feedback accountability via feedback utility in physicians.*
- *Hypothesis 2. Feedback value has a positive relationship with feedback accountability via feedback utility in physicians.*

### Change discrepancy as an antecedent of feedback value

*Change discrepancy* refers to individuals’ awareness of a situation that requires change (perceived need for change), which suggests a sense of relative prioritization and urgency [36]. Prior studies have demonstrated the crucial role of *change discrepancy* as a precursor to initial *readiness for change* and the corresponding change-related *actions* (i.e., initiation, persistence, and cooperative behaviours) [37–39]. A high sense of *change discrepancy* (derived from both perceiving *discrepancy* and evaluating such *discrepancy* as of high importance) triggers individuals’ information processing to understand the problem and explore opportunities for change [40]. Although it lacks empirical evidence in the feedback orientation literature, the logic of this argument remains relevant: individuals must perceive the need for change before starting to consider feedback as a candidate solution to initiate behavioural change, developing positive feedback orientation based on cognitive appraisals that lead to a resulting action. As such, we integrate it as an antecedent of the cognitive appraisal of *value* in the following hypothesis:

- *Hypothesis 3. Change discrepancy has a positive relationship with feedback value in physicians.*

**Fig. 1:**
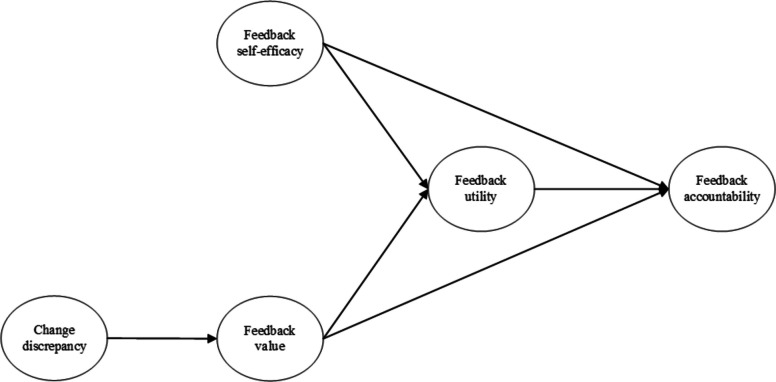
Integrated conceptual model of the recipient characteristics that influence engagement with Clinical Performance Feedback

Together, the above hypotheses form the basis of the resulting conceptual model (see Fig. 1).

## Methods

### Study design

We used a cross-sectional, multi-method approach to develop a model of physician attitudes and beliefs relating to CPF and to explore variation across key constructs. We achieved this in three sequential steps: (1) validate the model of engagement with CPF; (2) quantitatively explore recipient heterogeneity across key constructs; and (3) qualitatively explore recipient heterogeneity across key constructs. The protocol was approved by the Trillium Health Partners Research Ethics Board (ID # 1073). All participants provided informed consent prior to survey completion and interviews.

### Context and setting

This study was conducted in the primary care setting. In Ontario, Canada’s most populous province, most of the population (83%) has a primary care physician (PCP) as their first point of contact within the healthcare system [41], with approximately 14,500 physicians providing primary care services as of 2021 [41–43]. PCPs may work in various practice settings, such as solo practices, group practices, or Community Health Centres, and have different remuneration schedules available to them, ranging from fee-for-service to capitation to salary-based. Most PCPs in Ontario are paid through a blended capitation model, where they receive a fixed amount of money per patient registered to their practice based on factors such as age, sex, and health status (similar to capitation) as well as additional payments for specific services performed (similar to fee-for-service) [44, 45].

There are numerous CPF initiatives in Ontario that are specific to primary care physicians, including organizational initiatives [12] and system level initiatives related to antibiotic prescribing [46] and team-level improvement [47]. The most longstanding CPF report was introduced in 2013 and made available to all primary care physicians in Ontario who voluntarily registered. It was initially developed by Ontario Health in partnership with the Association of Family Health Teams of Ontario, the Association of Ontario Health Centres, and the Ontario College of Family Physicians. The individuals involved in developing the original report were members of regulatory organizations, working primarily at the system level, knowledgeable of populational health–related data, and familiar with these types of initiatives. At the time of the study, the report used administrative data sources to assess a series of quality indicators: safe prescription (eg, opioid and benzodiazepine prescription rates), cancer screening (eg, percentage of patients with up-to-date cancer screening tests for cervical, breast, and colon cancer), diabetes management (eg, percentage of patients with diabetes who had had ≥2 HbA1c tests within the past 12 months, who had diabetes and were aged >65 years and had an active statin prescription, and who had had a retinopathy screening test within the previous year), and health service use (eg, emergency department visits, hospital admissions, and readmissions [by condition]). Aggregate, practice-level data were presented for each of the indicators, covering the previous 12 months of clinical practice, and include peer comparators to help identify opportunities for improvement. Practice improvement ideas specific to each of the topics were included to support recipients in taking action. The CPF report is confidential and is not used for performance management, and there is no direct compensation for performance on these metrics. A full mock report from 2020 is included in Additional File 1. Prior work reported on the usability of this specific report, including suggestions for improvement [48].

### Step 1: model validation

#### Participant recruitment

Eligible participants included all registered PCPs (i.e., specialty of family medicine or general practice) in Ontario, Canada (*n* ≅ 14,700 as of 2018) [49]. Recruitment occurred via an online survey distributed via three channels: (1) the mailing lists of key organizations in primary care, (2) a targeted email from Ontario Health, the agency that oversees healthcare administration in the province of Ontario, to registered recipients of their MyPractice: Primary Care reports, and (3) social media channels (i.e., X). All recruitment materials provided a direct link to a letter of information and a subsequent electronic survey for those who consented. Survey participants were given the option to receive a $50 gift card for completing the survey.

#### Data collection

The electronic survey included constructs from two validated and widely adopted scales: the Feedback Orientation Scale (FOS) and the Organizational Change Recipients’ Beliefs Scale (OCRBS). The FOS [17, 18] measured feedback accountability, feedback self-efficacy, and feedback utility. We omitted the social awareness construct the items reference sensitivity to others’ opinions and making a good impression. CPF includes objective data on patient outcomes and is delivered privately to physicians. CPF reports include comparative data but do not convey others’ opinions or reputational judgments. The OCRBS [36, 50] measured perceptions on feedback value and change discrepancy. All items were measured on a 5-point Likert scale ranging from “strongly disagree” to “strongly agree”. To better align with the context of survey participants, we modified the original OCRBS items to contextualize statements around the need for change (i.e., *change discrepancy*) and perceived intrinsic and altruistic value that would come with receiving feedback (i.e., *feedback value*) in the healthcare field. An overview of constructs and definitions can be found in Table 1.

**Table 1:** Survey constructs measuring recipient characteristics

**Construct of interest**	**Source Survey**	**Operational Definition**
Feedback utility	FOS	The belief that CPF will improve the desired outcomes
Feedback self-efficacy	FOS	Confidence in the ability to act on CPF
Accountability	FOS	The sense of responsibility for acting on CPF
Change discrepancy	OCRBS	The belief that something needs to improve
Value^a^	OCRBS	Whether CPF provides value in line with the perceived discrepancy

*CPF* Clinical perfromance feedback, *FOS* Feedback Oreintation Scale, *OCRBS* Organizational Change Recipients’ Beliefs Scale

^a^Referred to as valence in the OCRBS.

#### Data analysis

Common method bias was assessed via multi-collinearity [51] and goodness-of-fit [52] and Confirmatory Factor Analysis was conducted to test the discriminant validity of the study constructs [53]. Partial Least Squares Path Modeling (PLS-PM) was then applied to test the conceptual model due to its capability of investigating complex latent construct models using a small sample size with non-parametric data [54, 55]. The PLS-PM model was estimated to measure the direct effects, indirect effects, and total effects of the included constructs. Missing values were replaced by the mean value. To test the statistical significance of the PLS-PM estimates and provide the corresponding confidence intervals, a bootstrapping procedure based on 5000 resamples was applied [56]. Mediation analysis followed Zhao, Lynch and Chen [57]. All statistical analyses were performed in R 4.4.1, using packages including tidyLPA, lavaan, nnet, SEMinR, and plspm.

#### Results

After data cleaning the survey had 206 responses (see Table 2 for participant demographics). Results of all construct level Variance Inflation Factor scores were under the threshold of 3, indicating absence of multi-collinearity [56]. Goodness-of-fit was 0.49, higher than the cut-off of 0.36 to be considered as globally fit [58]. The original model yielded a satisfactory model fit to the sample data [54, 59, 60], including: Comparative Fit Index (CFI) > 0.90, Tucker–Lewis Index (TLI) > 0.90, Root Mean Square Error of Approximation (RMSEA) < 0.08, Standardized Root Mean Squared Residual (SRMR) < 0.08, and scaled χ2/degree of freedom < 3 (see Additional File 2). Moreover, the original model had better model fit than the reduced models in all criteria. These analysis results indicated that the Common Method Bias was not an issue. After removing two items that did not meet the indicator loading threshold of 0.6 [61], the model demonstrated acceptable internal consistency, construct validity [62], and discriminant validity [56, 63] (refer to Additional File 3 for detailed results).

**Table 2:** Participant Characteristics

F	n	%
Gender		
Man	78	38.8
Woman	123	61.2
Missing	5	
International graduate		
No	166	81.4
Yes	38	18.6
Missing	2	
Practice year		
≤5 years	62	30.1
≥6 years	144	69.9
Work days/week		
≤ 3 days	77	37.4
≥4 days	129	62.6
Practice model		
Family Health Organization (FHO)	92	44.7
Other	114	55.3
Community size		
Urban centre	94	45.6
Smaller areas	112	54.4
Staff number within the organization		
≤5	95	46.1
≥6	111	53.9

**Fig. 2:**
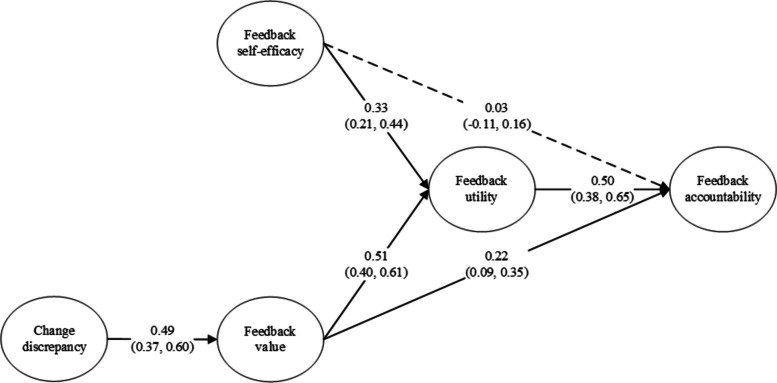
Estimated relationships within the integrated model. N.B. Numbers in parentheses indicate 95% confidence intervals. Dashed arrows indicate effects that are not statistically significant

Figure 2 presents the integrated model and Table 3 presents means, standard deviations, and inter-correlations of the study constructs.

**Table 3:** Means, Standard Deviations, and Inter-Correlations of Study Constructs

	Mean	SD	C1	C2	C3	C4	C5
Change discrepancy (C1)	4.13	0.65	1				
Feedback value (C2)	3.66	0.60	0.49	1			
Feedback self-efficacy (C3)	3.72	0.61	0.20	0.35	1		
Feedback utility (C4)	3.90	0.60	0.48	0.62	0.51	1	
Feedback accountability (C5)	3.89	0.52	0.38	0.55	0.37	0.66	1

- *Hypothesis 1: Feedback self-efficacy has a positive relationship with feedback accountability via feedback utility.*

As Fig. 2 shows, we observed a significantly positive association between *feedback self-efficacy* and *feedback utility* (*β* = 0.33, 95% CI: 0.21, 0.44), between *feedback utility* and *feedback accountability* (*β* = 0.50, 95% CI: 0.38, 0.65), but no significant association between *feedback self-efficacy* and *feedback accountability* (*β* = 0.03, 95% CI: −0.11, 0.16). Taking into consideration the significant indirect effect of *feedback self-efficacy* on *feedback accountability* via *feedback utility* (*β* = 0.17, 95% CI: 0.10, 0.25), the mediation analysis revealed a full mediation effect of *feedback utility*, supporting Hypothesis 1.

- *Hypothesis 2: Feedback value has a positive relationship with feedback accountability via feedback utility.*

We observed a statistically significant positive association between *feedback value* and *feedback utility* (*β* = 0.51, 95% CI: 0.40, 0.61) and between *feedback utility* and *feedback accountability* (*β* = 0.50, 95% CI: 0.38, 0.65). The mediation analysis yielded a significant indirect effect of *feedback value* on *feedback accountability* via *feedback utility* (*β* = 0.26, 95% CI: 0.17, 0.36). Considering a significant direct effect of *feedback value* on *feedback accountability* (*β* = 0.22, 95% CI: 0.09, 0.35), the result indicated a complementary mediation effect of *feedback utility*, supporting Hypothesis 2.

- *Hypothesis 3: Change discrepancy has a positive relationship with feedback value.*

We observed a statistically significant positive relationship between *change discrepancy* and *feedback value* (*β* = 0.49, 95% CI: 0.37, 0.60), supporting Hypothesis 3.

### Step 2: Quantitatively explore recipient heterogeneity

#### Participant recruitment and data collection

The same participants and electronic survey data described in Step 1 were used.

#### Data analysis

We conducted a Latent Profile Analysis (LPA), a novel person-centered approach [64] not yet applied in the context of CPF, to identify the unique participant profiles that contribute to population heterogeneity in feedback orientation (i.e., *feedback self-efficacy*, *feedback utility*, and *feedback accountability*). Confirmatory factor analysis (CFA) was used to validate the assumptions that the identified latent models were distinct and reliable [65, 66]. To address non-normality and missing values, we used Maximum Likelihood with Robust standard errors estimation and Full Information Maximum Likelihood estimation, respectively. We extracted the factor scores of each construct following Scherer et al. [66] to generate more accurate estimates for each latent construct.

#### Results

After data cleaning the survey had 206 responses (see Table 2), with the majority being women (61.2%), Canadian trained physicians (81.4%), with more than 5 years of practice experience (69.9%). Participants reported working more than 3 days/week (62.6%), with more than 5 colleagues (53.9%). Nearly half of the participants (45.6%) were based in an urban centre (see Table 2 for survey participant demographics).

**Fig. 3:**
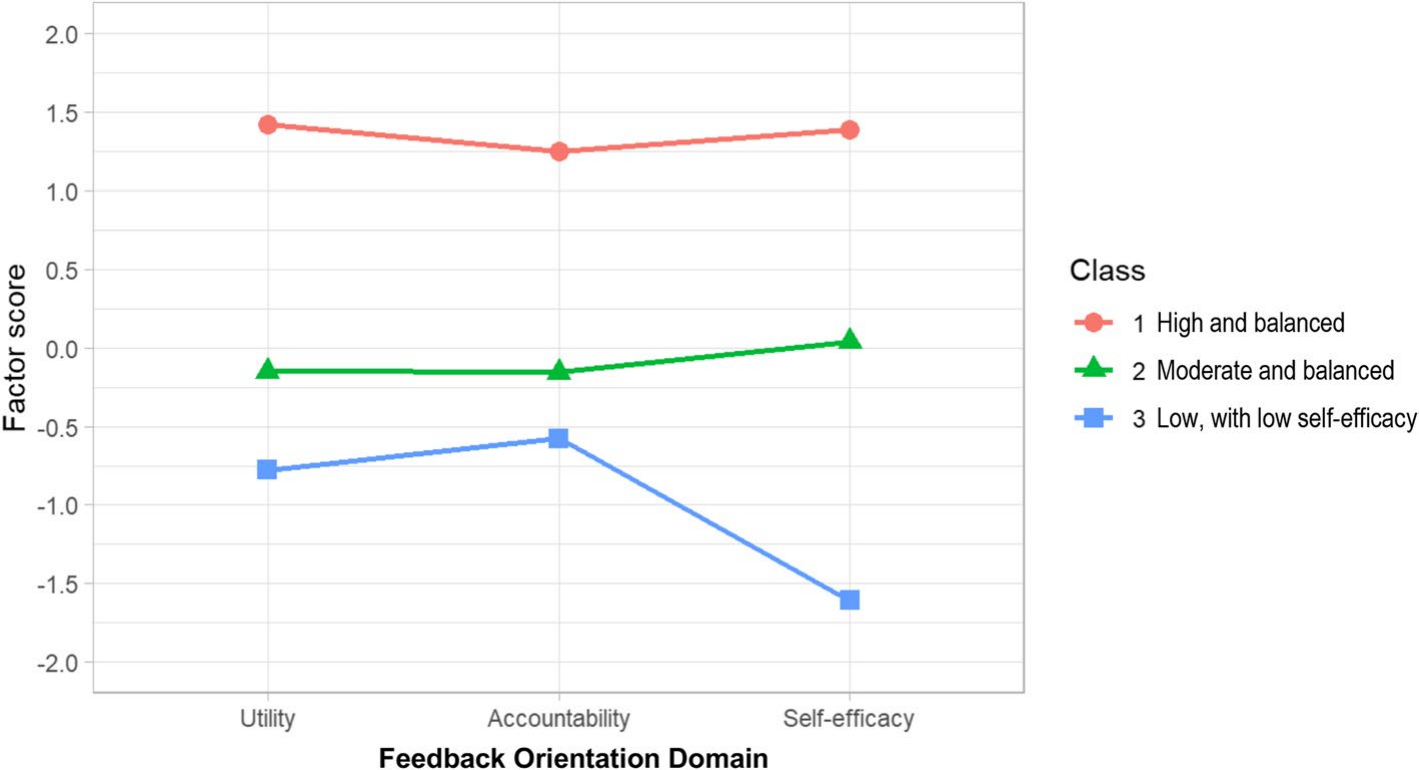
Latent profiles of feedback orientation among primary care physicians

LPA identified three distinct class profiles (see Additional File 4), assigning 32 participants to Class 1, 143 participants to Class 2, and 31 participants to Class 3. Participants in Class 1 exhibited consistently higher factor scores than 0 (i.e., overall sample mean) on all profile indicators; those in Class 2 exhibited factor scores around 0 on all profile indicators; whereas those in Class 3 exhibited factor scores lower than 0, especially for *feedback self*-efficacy (see Fig. 3). In this case, we labelled these three classes as “high and balanced feedback orientation”, “moderate and balanced feedback orientation”, and “low feedback orientation (especially *feedback self-efficacy*)”, respectively.

Table 4 reports the association of demographic and psychological characteristics with feedback orientation class membership. Using Class 2 as a reference group, we found that *feedback value* and *change discrepancy* were positively associated with the likelihood of being assigned to Class 1, e.g., for every one-unit increase in *feedback value*, the odds of being assigned to Class 1 increased by 508% compared to being assigned to Class 2. *Feedback value* was negatively associated with the likelihood of being assigned to Class 3 compared to being assigned to Class 2. Additionally, women were more likely to be assigned to Class 3, whereas those who worked in a practice model other than FHO were less likely to be assigned to Class 3 as compared to being assigned to Class 2.

**Table 4:** Demographic and Psychological Characteristics Predicting Profile Membership of Feedback Orientation

	Class 1 vs Class 2			Class 3 vs Class 2		
	OR	95% confidence interval	p	OR	95% confidence interval	p
Feedback value (OCRBS)	6.08	2.11, 17.55	0.001	0.28	0.12, 0.67	0.005
Change discrepancy (OCRBS)	3.81	1.30, 11.20	0.015	1.62	0.77, 3.42	0.199
Gender (Woman vs Man)	0.49	0.18, 1.30	0.149	5.11	1.56, 16.71	0.007
International graduate (Yes vs No)	0.64	0.19, 2.19	0.477	1.63	0.46, 5.79	0.446
Practice year (≥6 vs ≤5)	1.15	0.37, 3.58	0.805	1.58	0.59, 4.27	0.363
Work days/week (≥4 vs ≤3)	1.16	0.39, 3.45	0.784	0.97	0.37, 2.55	0.945
Practice model (Other vs FHO)	0.70	0.26, 1.87	0.475	0.30	0.12, 0.77	0.013
Community size (Smaller size vs urban)	0.58	0.22, 1.52	0.266	0.85	0.34, 2.13	0.734
Staff number (≥6 vs ≤5)	0.39	0.14, 1.06	0.064	0.97	0.38, 2.48	0.952

N.B. Class 1: high and balanced feedback orientation, Class 2: moderate and balanced feedback orientation, Class 3: low feedback orientation (especially *self-efficacy*).

### Step 3: Qualitatively explore recipient heterogeneity

#### Participant recruitment

We used a convenience sampling approach for the interviews by including a question at the end of the electronic survey asking participants if they were interested in participating in a follow-up interview to discuss their beliefs and perspectives relating to CPF. Participants were advised they would be provided with a $150 honorarium for interview participation. We aimed to recruit PCPs eligible to receive the CPF report, whether they signed up for it or not. We contacted interested participants in consecutive order (i.e., starting with those that completed the survey first). We continued recruitment until no new insights emerged from the interviews.

#### Data collection

A convenience sampling approach operationalized through a question at the end of the online survey generated a list of eligible participants for the interviews. Semi-structured, exploratory interviews were informed by the survey constructs (Table 1) and sought to understand participant perspectives on CPF (see Additional File 5 for interview guide). Questions explored perspectives and beliefs relating to performance improvement in primary care, participant goals around quality of care, recipient and contextual factors perceived to influence engagement with CPF, and experiences with CPF. Example questions included ‘What role does feedback play for primary care physicians and the care they provide’ and ‘How does feedback contribute to your success (or not) as a primary care physician?’. As the focus of this study was on recipient attitudes and characteristics, data on contextual factors will be reported separately. Interviews were conducted virtually and were audio-recorded and transcribed verbatim by an independent third party.

#### Data analysis

Transcripts were coded using MAXQDA. Interviews were analyzed using the framework method [67, 68], with survey constructs and CP-FIT domains applied as pre-defined deductive codes. Inductive coding was applied when concepts were identified that did not fit within the definitions of pre-defined constructs. This approach supported exploration of potential interactions between individual attitudes and beliefs that may influence engagement with CPF. Once descriptive statements were generated to detail how each construct manifested in the data, we reviewed the transcripts to identify how these characteristics intersected with one another to generate themes. To support the framework analysis, qualitative findings on recipient factors influencing engagement were triangulated with participant survey results to achieve a deeper understanding of how these factors coalesce to shape the perceptions and beliefs of physicians [69]. This triangulation helped identify the characteristics (i.e., distinct attitudes and beliefs) that varied between individuals, enabling the development of typologies for participants who recognized the value of CPF and those who did not. These insights support the refinement of the conceptual model.

#### Results

Nine interviews were conducted with PCPs, ranging from 40 minutes to 62 minutes in duration (see Table 5 for participant demographics and survey scores). Two distinct viewpoints emerged – one where participants did not see value in CPF, emphasizing its limitations and lowering perceived accountability (hereafter referred to as non-engagers), and one where participants acknowledged the limitations of CPF but still believed in its value and their accountability to improve (hereafter referred to as engagers). Simply put, some participants self-reported limited use of the report *because of* its limitations (*n*=5) while others perceived it as useful *despite* its limitations (*n*=4). Four key perceptions distinguished these groups, which are described below in line with the constructs described above.

**Table 5:** Interview Participant Survey Scores and Practice Demographics

**Study ID**	**FOS**			**OCRBS**				**Personal characteristics**		**Practice**			
	Utility	Accountability	Self-Efficacy	Average	Value^a^	Support	Discrepancy	*Gender*	*International graduate*	*Practice years*	*Work days/week*	Primary Funding Model	Number of Staff
Non-engagers													
1	3.2	3.4	3.8	2.9	2.0	3.3	3.3	Man	No	≥6 years	≥4 days	FFS	≤5
2	2.6	3.8	2.4	3.3	2.5	3.0	4.5	Woman	No	≥6 years	≤3 days	FFS	≥6
3	3.6	3.8	3.6	3.0	3.0	3.2	2.8	Woman	No	≥6 years	≥4 days	FFS	≤5
6	3.2	3.2	2.2	3.4	4.3	2.0	5.0	Man	No	≥6 years	≤3 days	CAP	≥6
12	3.2	3.6	3.6	2.4	2.3	2.5	2.5	Man	No	≥6 years	≥4 days	FFS	≤5
Engagers													
7	4.8	5.0	4.4	3.8	3.8	3.7	4.0	Woman	No	≥6 years	≥4 days	FFS	≥6
9	3.8	4.2	3.2	3.6	3.5	4.0	3.3	Woman	No	≥6 years	≥4 days	FFS	≤5
10	4.2	4.0	3.8	4.1	4.3	4.0	4.3	Man	No	≥6 years	≥4 days	CAP	≥6
15	4.0	4.2	3.8	4.5	4.3	4.3	5.0	Woman	No	≥6 years	≥4 days	CAP	≤5

*CAP* Capitation payment, *FFS* Fee for service

^*a*^*Measured via the valence domain in the OCRBS*

### Perceived need for change and value of CPF

Physicians unanimously recognized the need for change within the primary care landscape, citing administrative burdens, deferred care due to the pandemic, and the departure of primary care physicians from the profession as factors that drive physician burnout (and therefore necessitate change). Despite a shared acknowledgement of the need for change, participants held varying perspectives on the role of CPF in informing change. Engagers tended to view CPF as a catalyst, actively considering themselves among those responsible to drive some of these changes. Conversely, non-engagers downplayed the role of CPF, emphasizing changes outside their professional scope as more impactful.

*“It’s like, patients are already getting reminders from [a regional support organization] for all three of these, breast, colon, cervical. Patients can book their own mammograms. Ideally, if I was going to change anything, why doesn't the province mail colon cancer screening kits directly to patients, instead of me having to tell the labs to do it? Some of these things are like, you’re telling me these numbers, so what? So I can call the patients and remind them they got a letter in the mail? Is that what you want me to do? Because you already have [the regional organization] doing this stuff, and now you’re asking me to do it as well.” P6, Non-Engager*

These varying perspectives aligned with participant scores on the OCRBS. While engagers and non-engagers scored similarly on the ‘Discrepancy’ factor, which measures the extent to which one feels that there are legitimate needs for change, non-engagers scored lower on the ‘value’ factor, which measures the perceived importance of CPF as an improvement strategy, including the extent to which recipients believe that they will benefit from engagement with it. As highlighted above, the assessment of value is influenced by the recipient’s professional context as well as the overarching system context, including available resources and supports. Importantly, non-engagers stressed that their criticism of CPF was not directed at the general practice of collecting data to inform practice, but rather at its implementation within currently available reports providing feedback at the practice level. Notably, lower value ratings among non-engagers reflected these implementation and context beliefs (e.g., that screening outreach sits with system programs rather than individual practices or the resulting action is someone else’s job), rather than rejection of CPF in principle.

### Perceived utility of CPF for addressing practice-related gaps

Engagers perceived the report was helpful in supporting them in their role, including general improvement and identifying practice-related gaps and setting and monitoring goals aimed at addressing them. One physician explained:

*“There’s this quality improvement project we have to do every five years, so I believe that might have been part of the impetus and […] use this as one avenue in which to explore how we’re going to improve from a quality improvement lens.” P10 (Engager)*

Non-engagers were less likely to perceive this benefit, which was supported by their lower scores on the ‘Utility’ domain of the FOS. Many non-engagers expressed having alternative ways of identifying practice-related gaps, such as leveraging other sources of data-driven feedback (e.g., system level reports that identified specific patients that need action) or querying their electronic medical record (EMR) system. In fact, most non-engagers claimed that physicians should already have a sense of their performance relative to provincial averages, leading them to believe the report didn’t provide any novel insight.

*“I think [the CPF report] said I had more than the average opioid patients. I could have told them that. I run searches on my patients. We’ve done audits to look for people who are on benzodiazepines, or higher doses of opioids. I have a list that I go through every few months. So, it’s less helpful for me personally because I’m already on top of the things that I can track and change.” P1, Non-Engager*

Additionally, some non-engagers believed that CPF reports were primarily targeting low performers—a belief that stemmed from perceptions regarding the goal of CPF reports. Non-engagers perceived the reports as a way of standardizing practice patterns, increasing adherence to clinical guidelines, or reducing healthcare costs. These goals were viewed as distinct from facilitating practice improvement and conflicted with the way some physicians viewed their role.

*“I was thinking to myself, why would [a regional organization] generate these reports? What’s it for? And ultimately, I’m like, ‘Oh, it’s because they want to save healthcare dollars.’ Which is, ultimately, a good thing, right? […] But the guidelines from [the regional organization] are based on populations. When I practice, I’m only thinking of the patient in front of me. I’m rarely thinking of the population. So, if you say to someone, “I’m recommending a FIT test, but actually a colonoscopy is better. We just can’t afford to do it for everybody.” They’re going to want the colonoscopy, right? They don’t care that [FIT tests are] more economical for Ontario at a population level, and nor should I, to some degree.” P2, Non-Engager*

### Perceived utility influences sense of accountability for engaging with and acting on CPF

Engagers and non-engagers held varying perceptions regarding the level of accountability they feel for engaging with and acting upon CPF, which aligned with scores on the ‘Accountability’ domain of the FOS. Engagers often described accountability as linked to comprehensive care.

“*There’s very little time, and there’s a lot of administrative duties. But part of my responsibility is to make sure I’m delivering high-quality care and trying to improve. So, I wouldn't say it’s extra admin stuff that I don’t have time for. I mean, to a degree, we have to make time. That’s part of the comprehensive care we're giving.” P9, Engager*

Two key factors emerged as shaping participants’ perceptions of accountability: the actionability of the feedback within the report and the broader organizational setting in which they worked.

In terms of actionability, participants unanimously viewed CPF related to preventative cancer screening and hemoglobin A1C testing as more actionable compared to feedback related to opioid and antibiotic prescribing. Engagers described *directional actionability*—using practice-level patterns to decide where to look next (e.g., targeted chart audits or discussions at team huddles)—rather than *operational actionability* (patient-level prompts for immediate intervention). Physicians viewed outside providers, such as pain clinics and walk-in clinics, as confounding opioid and antibiotic prescribing numbers, reducing participants’ sense of control and causing some to dismiss the data. This issue is compounded by the fact that practice-level CPF reports do not identify which patients have been prescribed these drugs and, unlike for preventative cancer screening, such information cannot be readily queried.

*“If I'm not able to tease out who it’s reflective of, in terms of actual patients, then it’s not useful. Because I actually don’t know. Let’s say, for example, it came back with something astronomically high, like 80 percent of your patients are prescribed opioids. OK, what’s actionable based on that? I guess I can ask patients more often if they're being prescribed opioids by other providers, but I can't affect other providers’ prescribing habits.” P6, Non-Engager*

Engagers were still more likely to find these indicators valuable, taking further lengths to contextualize and reflect on the data in ways that non-engagers did not. For example, one participant considered whether certain demographic characteristics of their patient population, such as rurality and proportion of patients with labor-intensive jobs, could be an influencing factor in the high prescribing rates of opioids. Another participant considered making changes to their practice to encourage patients to discuss opioid use, potentially paving the way for adjustments to treatment plans and deprescribing. Another described reflecting on their practice patterns in the context of broader patient outcomes:

*“The section on antibiotics, which is a new section, what was my antibiotic initiation rate […]. In March was below average. And then I went above average again. So the number of antibiotic treatment episodes prescribed by me in the last six months was 16. […] They’re basically saying fewer antibiotics is better, but I would say the right number of antibiotics is better. It would be interesting to see how your hospitalization rate compares to your antibiotic prescribing rate. Because perhaps your antibiotics are preventing hospitalizations.” P15 (Engager)*

Simply put, engagers acknowledged the importance of contextualizing the report’s findings to derive insights while non-engagers wanted insights to be self-evident and readily actionable.

## Discussion

### Summary of findings

The results of this multi-method study provide insight into the factors that influence physician engagement with CPF and how they interact. Specifically, perceived change discrepancy (whether physicians think that something needs to improve), value (whether CPF provides value in line with the perceived discrepancy), self-efficacy (the physician’s confidence in their ability to act on CPF), utility (whether physicians believe CPF will improve the desired outcomes), and accountability (sense of responsibility for acting on CPF) influence engagement with CPF. These insights advance our understanding of what to target to address commonly cited engagement challenges with CPF [3, 70, 71], and enhances CPF theory (specifically CP-FIT) by providing complementary insights on which recipient characteristics engagement with CPF and how they interact. However, the way these constructs manifest varies across physicians, with individual differences shaping the degree to which CPF is perceived as beneficial or actionable. These results extend prior work by demonstrating that engagement is not automatic, but rather is influenced by physicians' interpretation of its relevance, usability, and their own capacity to act on it – and that these interpretations themselves vary across physicians.

### Comparison with existing literature

The perceived value of CPF hinges on the belief that things need to change and its relevance to daily practice [12, 72]. Our findings expand on best practice guidance for CPF [6] and build on prior work that has found physicians do not engage with CPF when they perceive no benefit [73] by specifying the factors that influence how physician’s assess benefit. Physicians must first recognize a need for change (*change discrepancy*) and view CPF as providing relevant, useful data. When these conditions are met, engagement is more likely. However, our analysis captures a single point in time, and further research is needed to understand how these perceptions evolve and what strategies effectively shift them. Our study extends beyond the focus on barriers to engagement with CPF to highlight actionable pathways to foster engagement by specifying high-value determinant to target.

Physician self-assessment is often inaccurate [74, 75], limiting opportunities for professional growth and emphasizing the need for external assessments to guide improvement. CPF provides a scalable way to help physicians identify habitual processes in their practice patterns. However, engagement is not uniform; physicians vary in their confidence (self-efficacy) and willingness to act on CPF, influenced by their past experiences, learning preferences, and perceived relevance of the data. Many physicians lack confidence (self-efficacy) in using such data effectively [12, 72], aligning with the results of our latent profile analysis which showcase the role of self-efficacy in shaping feedback orientation. To effectively support improvement, CPF initiatives must simultaneously mitigate concerns and increase confidence in acting on the data, helping physicians to interpret population-level data and apply it to individual patients [12, 72]. Kluger and Nir’s feedforward approach [76] may help by shifting the focus from critiquing past performance to guiding future actions, highlighting actionable next steps. This approach aligns with the NCF, emphasizing CPF’s benefits (e.g., growth and improvement) while reducing perceived judgment (concerns). By linking self-efficacy to engagement with CPF, our findings reinforce the importance of designing CPF strategies that explicitly build physician confidence and competence in data use, rather than assuming these exist at baseline.

### Implications for practice

CPF initiatives should be designed with key engagement factors in mind, and implementation efforts should proactively communicate how CPF meets these needs. For example, specifying how the data aligns to physician values, including aspirations relating to clinical care, communication goals, and protecting time with patients downstream [73, 77], may increase perceived benefit. Those designing CPF should consider the explicit cues that would support two steps: review (engagement) and use (practice change). Situational strength—the contextual cues that shape behavior—plays a key role in influencing whether physicians change course [78]. To address this, CPF programs might increase perceived value by aligning metrics with local goals and patient priorities; increase perceived usefulness by surfacing clearer actions in line with recipient values; support self-efficacy through simple, how-to resources and peer troubleshooting; and clarify accountability via explicit follow-up expectations and feedback loops. Additionally, our results distinguish directional actionability (CPF as an orienting signal that supports review, prioritization, and planning) from operational actionability (patient-level prompts for immediate action). The CPF discussed by our participants provides directional actionability and should prompt appropriate follow-up mechanisms (e.g., targeted chart review, clinical decision support, or structured improvement cycles). CPF initiatives providing practice-level data should pair it with downstream audit tools, decision support prompts, or clear actions for recipients to translate directional signals into responsible, patient-level actions.

Delivering CPF with future-oriented goals and actionable guidance can make it more constructive, reframing it as a learning opportunity rather than a critique. Combining this approach with facilitated coaching conversations can help physicians create concrete plans to act on CPF, closing the oft-cited gap between intention and action [12, 72, 79–81]. These conversations should cue physicians to identify potential changes and guide them to making those changes routine practice, ultimately enabling them to intuitively respond to CPF without relying on effortful interpretation amid competing demands [82]. Taken together, these strategies provide a structured and evidence-informed roadmap for improving CPF engagement to better position it for impact on clinical practice. Applying the model to a specific CPF program requires layering in report features (e.g., design, content, cadence, usability) and the professional and organizational context (e.g., links to incentives, integration with performance reviews, reputation, or public reporting). These contextual layers shape clinicians perceived value (importance/relevance) and perceived utility (usefulness/actionability) of CPF, representing important boundary conditions for translation to practice that sit outside the scope of this first phase of work. In line with this work and prior theory, researchers and implementers should assess these elements and how they impact the model’s pathway from belief - engagement – use.

### Limitations

Primary care physicians in Ontario, Canada receive numerous CPF reports from various sources, therefore it is possible that preconceived notions of what feedback is could have affected the measurement of the related constructs. Given that CPF may cue perceived evaluation by authorities [12, 83, 84], we lacked a validated measure of perceived external evaluation and assessment of the accountability climate in this context. Future work should explicitly examine perceived oversight and the overarching accountability climate in the context of CPF carries consequences and map clinicians’ mental models of feedback in healthcare to determine whether a new mental model is needed**.** This study intentionally established a report-agnostic model of clinicians’ beliefs as a necessary first step. The next phase is to specify and measure program-level boundary conditions—report usability, cadence, and accountability arrangements (e.g., pay-for-performance, reputational consequences) and test how they influence the belief - engagement - use pathway. Researchers and those designing CPF can build on this work by developing data-grounded personas that synthesize common belief patterns aligned with the profiles identified here (high-and-balanced, moderate-and-balanced, and low with low self-efficacy) to inform tailoring of CPF interventions. Additional work is needed to explore whether the findings in this study extend beyond the current sample (primary care physicians), including other medical specialties and clinicians who receive CPF. Generalizability may be moderated by care-setting and system features such as practice structure (e.g., solo vs group, ambulatory vs hospital), payment and incentive arrangements, team composition and role delineation, external accountability (e.g., performance review, public reporting) which may shift perceived value, utility, self-efficacy, and accountability in predictable ways. Given the widespread and increasing frequency with which data is provided to physicians, future work should explore whether and how these beliefs may systematically affect physicians’ judgements and decisions as it relates to using data more generally to identify and drive improvements in their practice should be explored. The results from this study can be used as the basis for interventional studies, including those that engage physicians in co-designing CPF, to support better specification of how to address value, utility, confidence, and accountability in practice.

## Conclusions

This study offers novel insights into the cognitive appraisal process that influences whether physicians voluntarily engage with CPF. The evidence-based design of CPF has the potential to support physician development with an emphasis on improving patient outcomes. Specification of the factors informing CPF appraisal support the personalization of performance feedback strategies – akin to personalizing care for the patient in front of you. Based on our findings, we propose that CPF initiatives explicitly state how CPF supports better patient outcomes, maintain a future-focus, and provide clear instructions about the behaviour(s) leading to improved outcomes. Recognizing that ‘intention is everything’ [73], CPF initiatives must clearly communicate how they support professional growth and improve patient outcomes in order to realize their promise of supporting health system improvement at scale.

## Supplementary Information

Additional file 1.

Additional file 2.

Additional file 3.

Additional file 4.

Additional file 5.

## Data Availability

The datasets generated and/or analyzed during the current study are not publicly available due to participant confidentiality, but may be available from the corresponding author on reasonable request and with appropriate ethical approvals.

## Abbreviations

CFI: Comparative Fit Index
CPF: Clinical Performance Feedback
CP-FIT: Clinical Performance Feedback Intervention Theory
FOS: Feedback Oreintation Scale
NCF: Necessity-Concerns Framework
OCRBS: Organizational Change Recipients’ Beliefs Scale
PCP: Primary care physician
RMSEA: Root Mean Square Error of Approximation
SRMR: Standardized Root Mean Squared Residual
TLI: Tucker–Lewis Index
