# Supplementary material for: The engagement equation: a model for understanding what drives voluntary physician engagement with data-driven clinical performance feedback
Source: Implement Sci Commun. 2025 Dec 11;7:8. doi: 10.1186/s43058-025-00819-5 (PMC12801449; doi:10.1186/s43058-025-00819-5)
Supplement: Supplementary file 1 — Additional file 1. [file 43058_2025_819_MOESM1_ESM.pdf]

Version Release: May 2023
PRIVATE AND CONFIDENTIAL

MyPractice
Primary Care

A tailored report for quality care

Sample FHT

Reporting Period: September 30, 2022

Release: May 2023

FHT Rurality Index of Ontario Score: Rural (40+)

FHT LHIN: Toronto Central

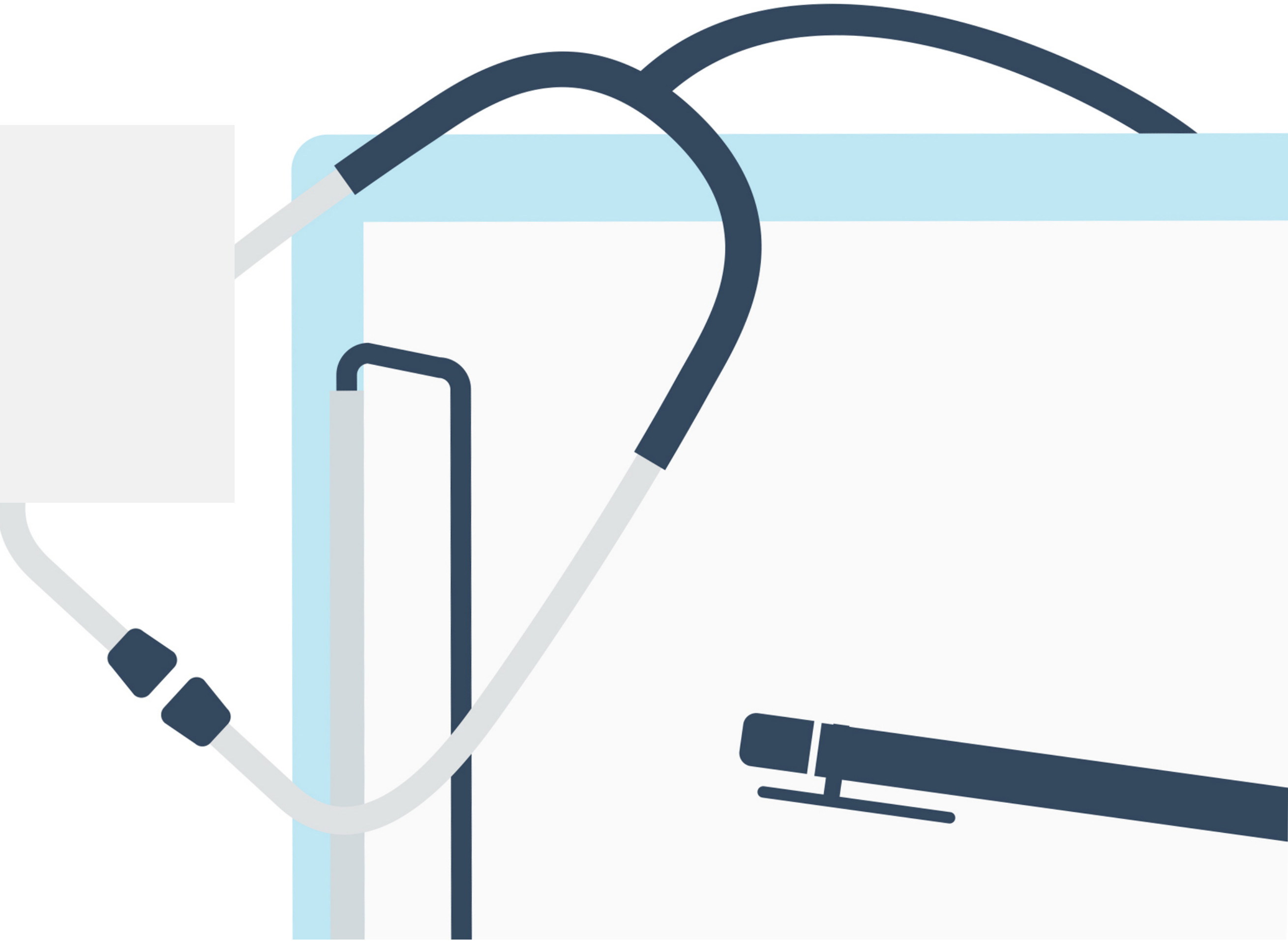

IC/ES
Ontario Health

Table of Contents

Report Overview **3**
Overall Indicators Summary **4**
Antibiotic Section **5**
Antibiotic Initiation ____ 5
Antibiotic Prolonged Treatment ____ 6
Quality Improvement Ideas for Antibiotic Prescribing ____ 7
Opioids Section **9**
Opioids Dispensed ____ 9
New Opioids Dispensed ____ 10
Opioids and Benzodiazepines Dispensed ____ 11
High-Dose Opioids Dispensed ____ 12
Quality Improvement Ideas for Opioid Prescribing ____ 13
Cancer Screening Section **15**
CRC Screening ____ 15
Pap Smear Screening ____ 16
Mammogram Screening ____ 17
Quality Improvement Ideas for Cancer Screening ____ 18
Diabetes Section **20**
HbA1c Testing to Prevent Complications from Diabetes ____ 20
Retinal Exam Testing to Prevent Complications from Diabetic Retinopathy ____ 21
Statins Dispensed to Prevent Vascular Complications from Diabetes ____ 22
Quality Improvement Ideas for Diabetes Management ____ 23
Health Service Utilization **25**
ED Visits ____ 25
Hospital Readmissions Within 30 Days ____ 25
SAMI Score ____ 25
Visits to Own Physician ____ 25
Visits to Own Group ____ 25
ACSC Admissions - Total ____ 26
Quality Improvement Ideas for Health Service Utilization ____ 27
My FHT's Patient Demographics and Disease Cohorts **28**
Methods and Acknowledgements **29**
References **32**

Ontario Health

Report Overview

Background

The *MyPractice:* Primary Care report can help you focus your quality improvement efforts.

This report DOES

- Use billing data and other administrative data.
- Give an overview of your practice activities.
- Provide the LHIN and provincial percentages for context.
- Provide you with ideas for improvement.
- Include rostered and virtually rostered patients.

This report does NOT

- Use EMR data held in your practice or provide direct links to your EMR.
- Provide details about specific patients.
- Include palliative care patients.
- Provide specific instructions for clinical care.
- Tell you what targets are best for your practice.

This report was developed by

Ontario Health and ICES in partnership with the Association of Family Health Teams of Ontario (AFHTO).

Additional information

- For more information about the *MyPractice:* Primary Care Report, please email us at PracticeReport@ontariohealth.ca.
- For more information on indicator definitions, please refer to the methodological notes on page 24. For more technical details, please refer to the technical appendix on the *MyPractice* web portal.

"I would strongly recommend that my colleagues sign up to receive this report. I think that it doesn't matter what model you are working in [...] this information tells you what you're doing as a family physician, and it tells you who you're looking after and how you're using the system"

- Dr. Cathy Faulds, Chief Clinical Lead, South West LHIN

Overall Indicators Summary

Data as of September 30, 2022

NEW

	**My FHT's Prescribing Rate is Higher Than Most of Family Health Teams** (higher than 60% of my peers)	**My FHT's Prescribing Rate is Around Average** (between 25th - 60th percentile)	**My FHT's Prescribing Rate is Better Than Most of Family Health Teams** (lower than 75% of my peers)
**Antibiotic Prescribing in my FHT** (pages 5-8)	Antibiotic Initiation<br>Antibiotic Prolonged Treatment	None	None

	**My Priority Indicators for Review** (below 40th percentile)	**My Indicators Around Average** (between 40th - 75th percentile)	**My Indicators Above Average** (above 75th percentile)
**Cancer Screening** (pages 15-19)	None	Pap smear testing	Mammogram testing<br>Any Colorectal screening
**Diabetes Management** (pages 20-24)	None	Retinal Exam testing	HbA1c testing

*Percentiles are based on FHTs registered for the *MyPractice:* Primary Care report

	# Patients Dispensed an Opioid	# Patients Newly Dispensed an Opioid	# Patients Dispensed an Opioid and Benzodiazepine	# Patients With a High-Dose Opioid >90 mg MEQ Daily
**Opioids Prescribing in my FHT** (pages 9-14)	**986**	**580**	**156**	**99**

Whom am I caring for?

Number of Patients

14,731

Age (mean)

46.1

Percent Male

48.5%

Percent Rural

55.3%

† Data suppressed where counts are between 1 and 5; additional suppression may be applied where counts are greater than 5 to prevent residual disclosure of suppressed values; N/A: Data not available; ¨ Please interpret with caution, denominator ≤ 30. For more details, refer to the Methods section on page 28.

Ontario Health

Antibiotic Section | Antibiotic Initiation

Data as of September 30, 2022

What was my FHT's antibiotic initiation rate for patients aged 66 and older?

- As of September 30, 2022, my FHT's antibiotic initiation rate was 102.8 per 1,000 encounters. My LHIN rate is 60.7.
- My FHT's antibiotic initiation rate is **higher than** the provincial rate of 74.1.

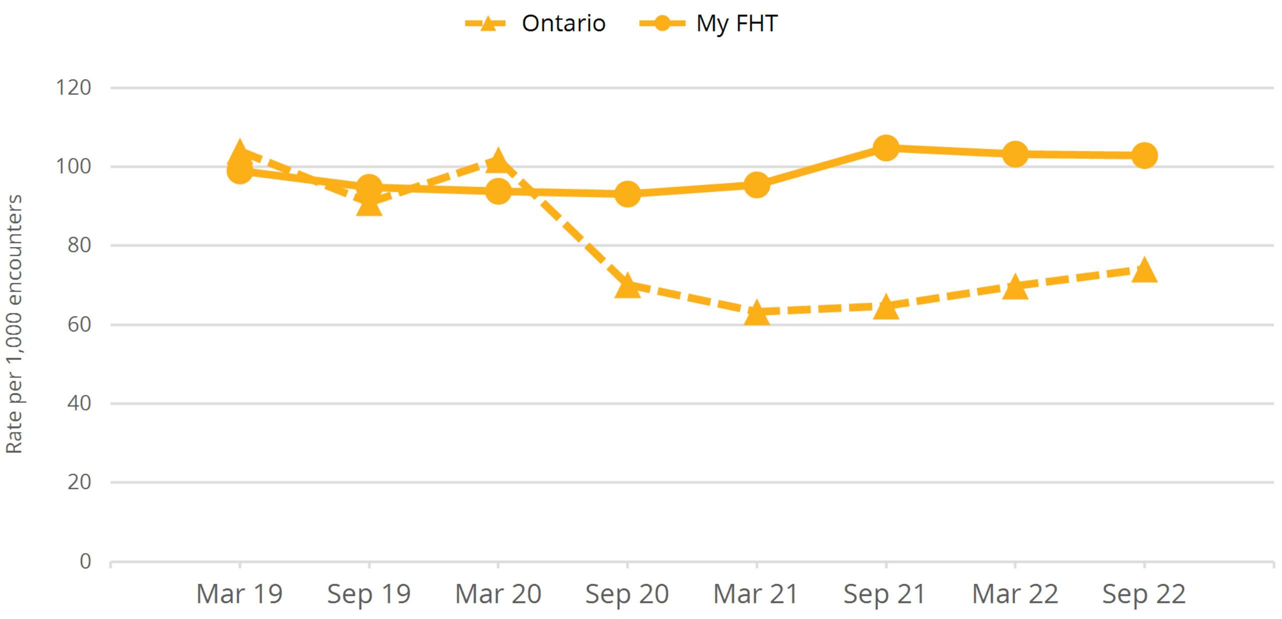

† Data suppressed where counts are between 1 and 5; additional suppression may be applied where counts are greater than 5 to prevent residual disclosure of suppressed values; N/A: Data not available; ¨ Please interpret with caution, denominator ≤ 30. For more details, refer to the Methods section on page 28.

Number of antibiotic treatment episodes prescribed by my FHT within the last 6 months
633

Did you know?

Most antibiotics in the community are prescribed by primary care physicians and 25 to 50 percent are likely unnecessary. (1-3) Primary Care physicians are key partners for antimicrobial stewardship.

Consider the benefit of using a viral prescription pad to encourage no antibiotics when appropriate.

Antibiotic Prolonged Treatment

Data as of September 30, 2022

What percentage of my FHT's antibiotic treatments were longer than 7 days in duration?

- As of September 30, 2022, 29.4% of my FHT's antibiotic dispenses were for longer than 7 days duration. My LHIN percentage is 25.7%.
- My FHT is **higher than** the provincial percentage of 25.7%.

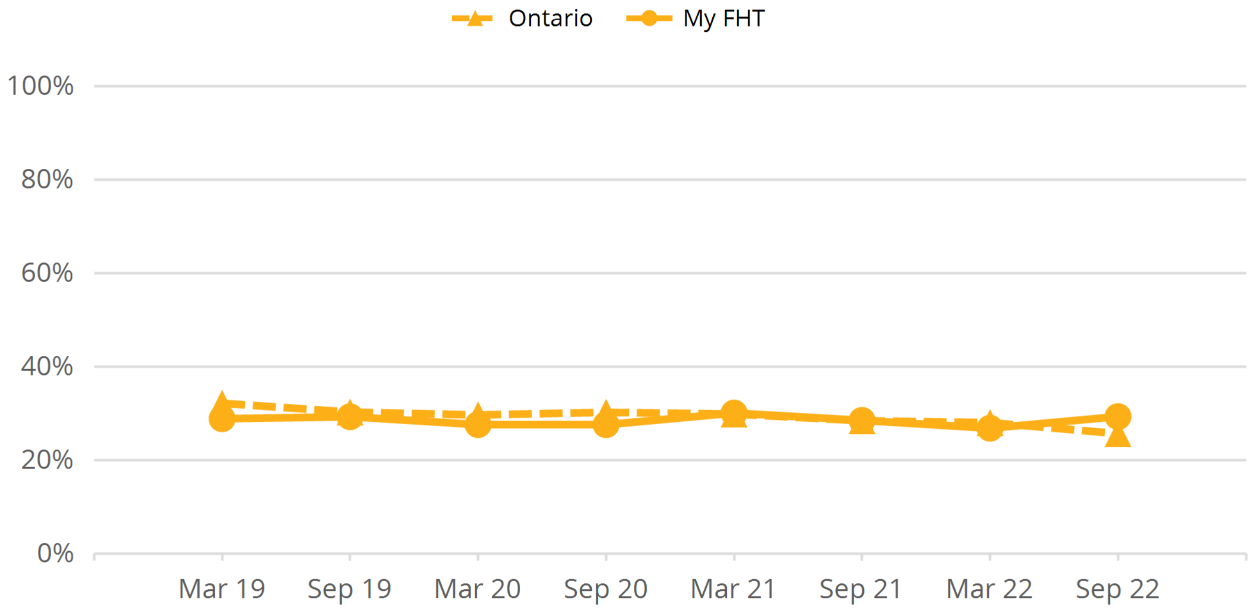

† Data suppressed where counts are between 1 and 5; additional suppression may be applied where counts are greater than 5 to prevent residual disclosure of suppressed values; N/A: Data not available; ¨ Please interpret with caution, denominator ≤ 30. For more details, refer to the Methods section on page 28.

Number of antibiotic treatment episodes longer than 7 days prescribed by my FHT, within the last 6 months
186

Did you know?

Shorter courses of antibiotics for common outpatient infections may result in fewer adverse events. (4-6)

Most community infections can be treated with 7 days of antibiotics or less. (7)

Ontario Health

Quality Improvement Ideas

How to Improve Antibiotic Prescribing in my Practice

Use the following steps to proactively practice antibiotic stewardship and guide improvement for safe antibiotic prescribing in your practice.

STEP 1: Identify opportunities for antibiotic stewardship

STEP 2: Where antibiotic stewardship is indicated, review treatment plans to identify gaps in the provision of evidence-based care

Identify Opportunities	Considerations for appropriate **INITIATION:**	If antibiotics **ARE** indicated: Considerations for appropriate **DURATION:**		If antibiotics **ARE NOT** indicated:
Identify patient encounters where there is an opportunity to optimize antibiotic use and prescribe more judiciously (common diagnoses listed below)	• *When are antibiotics indicated?*<br>• *Is it appropriate to initiate a course of antibiotics?*<br><br>Refer to Using Antibiotics Wisely in Primary Care (Choosing Wisely)	• *Did you select the appropriate drug and route?*<br>• *Is the duration of treatment the shortest effective evidence-based course of therapy?*	Tools and Resources to support antibiotic stewardship initiative for **DURATION:**	Tools and Resources to support antibiotic stewardship initiative for **INITIATION:**
• **Uncomplicated Otitis Media**	For vaccinated individuals aged >6 months (with either a perforated tympanic membrane with purulent discharge or a bulging tympanic membrane) with one of the following criteria: 1. Fever (**≥39°**C); 2. Moderately or severely ill; 3. Symptoms lasting > 48 hours	**5 days** (10 days in children <2)	General:<br>• Searchable database of Practice Guidelines - from the Association of Medical Microbiology and Infectious Disease Canada to review specific conditions where antibiotics are indicated as best treatment practices<br><br>Respiratory Tract Infections:<br>• The Cold Standard - Second Edition	Consider using tools such as Viral Prescription Pad and/or Delayed Prescription Pad to help educate patients on how to care for their illness without antibiotics<br><br>• See EMR instructions from Choosing Wisely on how to integrate these tools in your existing EMR<br><br>• Note: Delayed prescription pads are not recommended for conditions that never require antibiotics
• **Uncomplicated pharyngitis**	Patient's modified Centor score is ≥ 2 **AND** throat swab culture (or rapid antigen test if available) confirms presence of GAS	**10 days (depending on drug)**		
• **Uncomplicated sinusitis**	Symptoms persist for over 7 to 10 days. Has at least 2 of the PODS symptoms (one of those being O or D), **AND** has one of the following criteria: 1. severe symptoms; 2. mild to moderate symptoms with no response after a 72 hour trial with nasal corticosteroids	**5 days**		
• **Pneumonia**	CXR, if accessible, showing pneumonia (respiratory crackles alone during PE is not sufficient to establish a diagnosis)	**5 days**		
• **Acute exacerbation of COPD**	Clear increase in sputum purulence with either increase in sputum volume and/or increased dyspnea.	**5 days**		
• **Upper respiratory tract infection**<br>• **Influenza like illness**<br>• **Bronchitis / bronchiolitis / asthma**	No role for antibiotics	**No role for antibiotics**		
• **Cellulitis**	Rapidly spreading erythema, swelling, tenderness, and warmth, +/- lymphangitis and regional lymph node inflammation. Symptoms are typically unilateral	**5 days**		
• **Cystitis or lower UTI**	Symptomatic bacteriuria	**3-5 days** (depending on drug)		

Ontario Health

Quality Improvement Ideas

How to Improve Antibiotic Prescribing in my Practice

STEP 3: Explore opportunites for improvement

The following are common actionable themes and resources that can help support your quality improvement initiatives in safer antibiotic prescribing

Connect with peers and external supports	Access Continuing Education	Redesign Your System and Leverage Digital Health Solutions	Engage Your Patients
Contact a practice facilitator to help build capacity for improvement<br><br>• Quality improvement decision support specialists - AFHTO<br>• FHTs/NPLCs/CHCs: Resources are available on their Trello Board platform. Email improve@afhto.ca to sign up and gain access<br><br>Participate in practice coaching and peer group mentorship<br>• Peer Leaders - OntarioMD<br>• OMD Advisory Service - OntarioMD<br>• EMR Practice Enhancement Program - OntarioMD	Explore continuing professional development opportunities<br>• Antimicrobial Stewardship in Primary Care Continuing Education Program - University of Waterloo, School of Pharmacy<br>• Continuing Professional Development - Association of Medical Microbiology and Infectious Disease Canada<br>• Workshops/Webinars - Canadian Foundation for Infectious Disease<br><br>Learn about antibiotic stewardship<br>• Family medicine Antimicrobial Stewardship Campaign - Using Antibiotics Wisely<br><br>Include in quality improvement initiatives<br>• Consider addressing antibiotic stewardship and using your *My*Practice Primary Care Report to satisfy requirements for CPSO's Quality Improvement Program<br>• Access Quorum's Quality Improvement QI Tools and Resources from Ontario Health to support each step in your journey	Systematically re-evaluate and review evidence-based resources on appropriate intitiation and duration of antibiotic therapy<br><br>Implement supporting tools for Using Antibiotics Wisely in the Era of COVID-19 and Virtual Care<br>• The Cold Standard - Choosing Wisely<br><br>Develop and test a virtual care strategy to support patient care management<br>• Adopting and Integrating virtual visits into care - Ontario Health<br><br>Include the indication with each antibiotic prescription to improve medications safety and communication within the health team<br>• Randomized Clinical Trial - Journal of the American Medical Association<br><br>Measure your practice's antibiotic use to identify successes and opportunities for improvement<br>• Metrics for evaluating antibiotic use - JAC-Antimicrobial Resistance<br>• Quality Improvement Process Mapping - Ontario Health	Leverage Public Health Ontario's shared decision-making patient and prescriber resources<br>• Antimicrobial Stewardship in Primary Care: Let's Talk Antibiotics - Public Health Ontario<br><br>Engage patients in their care management<br>• Using Antibiotics Wisely Patient Resources - Choosing Wisely<br>• Patient Involvement in Decisions About Care - Quorum (Ontario Health)<br>• Patient Partnering Framework - Ontario Health<br><br>Review the Government of Canada's antibiotic resistance awareness materials<br>• For Patients<br>• For Healthcare Providers

Ontario Health

Opioids Section | Opioids Dispensed

Data as of September 30, 2022

What percentage of my FHT's non-palliative care patients have been dispensed an opioid prescription (excluding opioid agonist therapy) within the last 6 months?

- As of September 30, 2022, 6.7% of my FHT's patients have been dispensed an opioid prescription. 51.4% of those opioids were prescribed by the patients' physician within the FHT and 48.6% were prescribed by other providers (e.g., other family physicians, dentists, surgeons) within or outside of the FHT.
- My LHIN percentage is 4.8%. The provincial percentage is 5.3%. **These percentages are for context only and do not represent a target.**

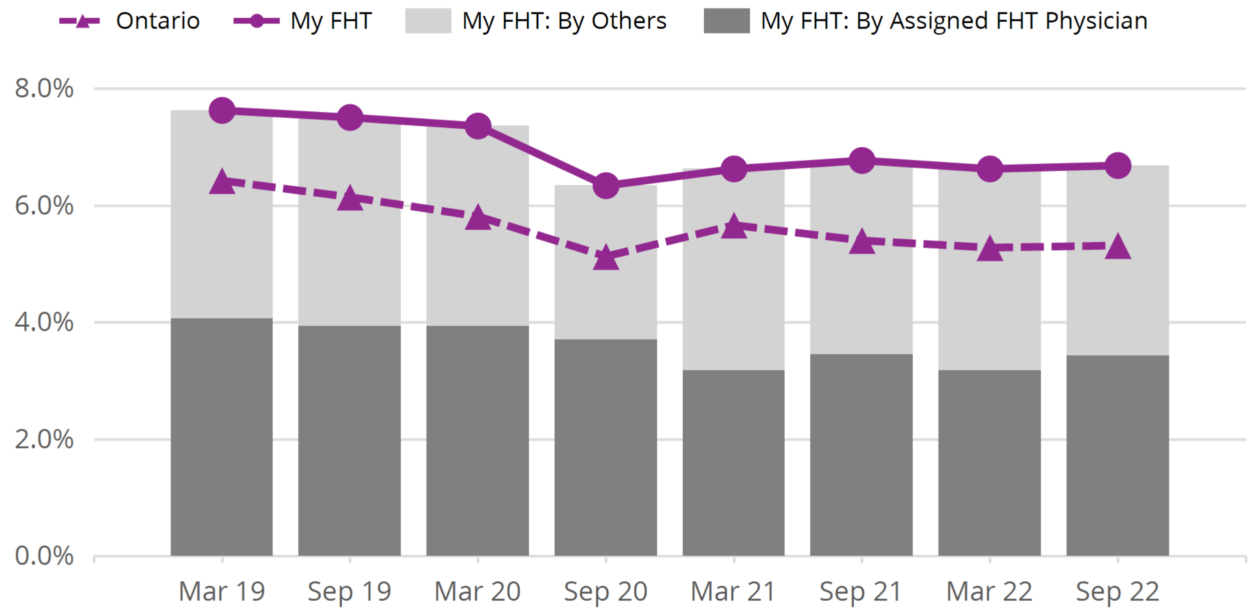

† Data suppressed where counts are between 1 and 5; additional suppression may be applied where counts are greater than 5 to prevent residual disclosure of suppressed values; N/A: Data not available; ¨ Please interpret with caution, denominator ≤ 30. For more details, refer to the Methods section on page 24.

Palliative care patients are not included; they were identified from hospital and physician billing claims data. For this indicator, the opioid medication's definition does not include opioid agonist therapy, opioid cough and anti-diarrheal medications.

**Number of my FHT's patients who have been dispensed an opioid within the last 6 months**
By their physician within the FHT: **505** By Other Providers within or outside of the FHT: **481**

Your patients who have pain need your primary care team.

Sometimes opioid prescriptions are appropriate. The data cannot weigh the benefits against the possible harms, but they can point to practice patterns worthy of reflection.

How many patients are taking opioids for a short-term acute use? Longer-term chronic use? (page 13)

Ontario Health

New Opioids Dispensed

Data as of September 30, 2022

What percentage of my FHT's non-palliative care patients have been newly dispensed an opioid prescription (excluding opioid agonist therapy) within the last 6 months?

- As of September 30, 2022, 3.9% of my FHT's patients have been newly dispensed an opioid prescription. 21.7% of those opioids were prescribed by the patients' physician within the FHT and 78.3% were prescribed by other providers (e.g., other family physicians, dentists, surgeons) within or outside of the FHT.
- My LHIN percentage is 3.1%. The provincial percentage is 3.2%. **These percentages are for context only and do not represent a target.**

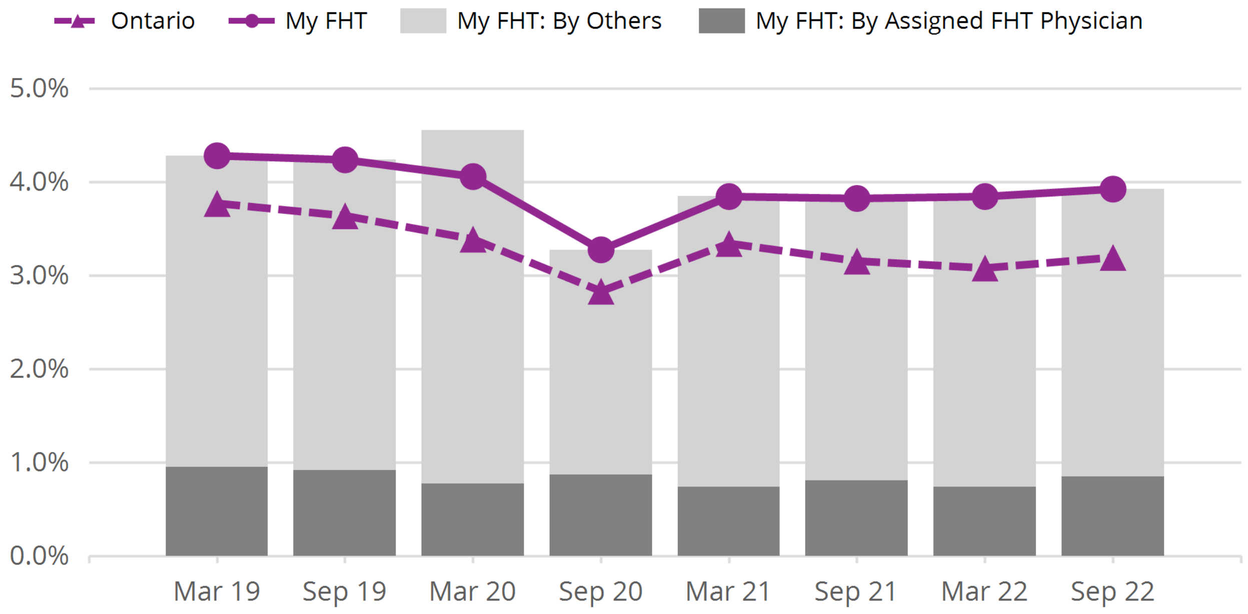

† Data suppressed where counts are between 1 and 5; additional suppression may be applied where counts are greater than 5 to prevent residual disclosure of suppressed values; N/A: Data not available; ¨ Please interpret with caution, denominator ≤ 30. For more details, refer to the Methods section on page 24.

Palliative care patients are not included; they were identified from hospital and physician billing claims data. For this indicator, the opioid medications' definition does not include opioid agonist therapy, opioid cough or anti-diarrheal medications.

Number of my FHT's patients newly dispensed an opioid within the last 6 months
By their physician within the FHT: **126** By Other Providers within or outside of the FHT: **454**

Your patients who have pain need your primary care team.

Sometimes opioid prescriptions are appropriate. The data cannot weigh the benefits against the possible harms, but they can point to practice patterns worthy of reflection.

How can we reflect on opioid prescribing patterns in our FHT? (page 13)

Ontario Health

Opioids and Benzodiazepines Dispensed

Data as of September 30, 2022

What percentage of my FHT's non-palliative care patients have been dispensed an opioid (including opioid agonist therapy) and benzodiazepine within the last 6 months?

- As of September 30, 2022, 1.1% of my FHT's patients have been dispensed an opioid and benzodiazepine. 66.0% of those opioids were prescribed by the patients' physician within the FHT and 34.0% were prescribed by other providers (e.g., other family physicians, dentists, surgeons) within or outside of the FHT.
- My LHIN percentage is 0.8%. The provincial percentage is 0.9%. **These percentages are for context only and do not represent a target.**

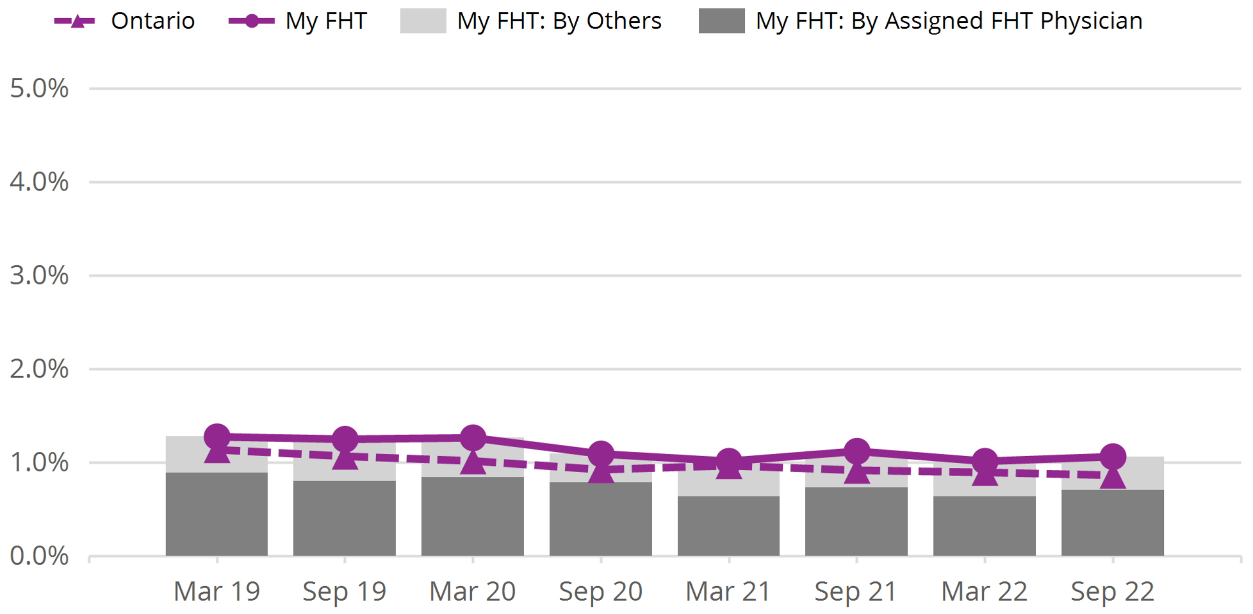

† Data suppressed where counts are between 1 and 5; additional suppression may be applied where counts are greater than 5 to prevent residual disclosure of suppressed values; N/A: Data not available; ¨ Please interpret with caution, denominator ≤ 30. For more details, refer to the Methods section on page 24.

Palliative care patients are not included; they were identified from hospital and physician billing claims data. For this indicator, the opioid medications' definition does not include opioid cough or anti-diarrheal medications.

**Number of my FHT's patients dispensed an opioid and benzodiazepine within the last 6 months**
Both by their physician within the FHT: **103**
One or Both by Other Providers within or outside of the FHT: **53**

Your patients who have pain need your primary care team.

The pharmacology suggests that sedatives and opioids enhance the depressant effect of the other, worsening the balance of harms versus benefits, though supporting evidence is unavailable. The expert perspective is that opioids and benzodiazepines should very rarely be prescribed together (8).

How can we reflect on opioid prescribing patterns in our FHT? (page 13)

Ontario Health

High-Dose Opioids Dispensed

Data as of September 30, 2022

What percentage of my FHT's non-palliative care patients have at least one high-dose opioid >90 mg MEQ daily within the last 6 months?

- As of September 30, 2022, 0.7% of my FHT's patients have a high-dose opioid >90 mg MEQ daily. 60.3% of those opioids were prescribed by the patients' physician within the FHT and 39.7% were prescribed by other providers (e.g., other family physicians, dentists, surgeons) within or outside of the FHT.
- My LHIN percentage is 0.4%. The provincial percentage is 0.5%. **These percentages are for context only and do not represent a target.**

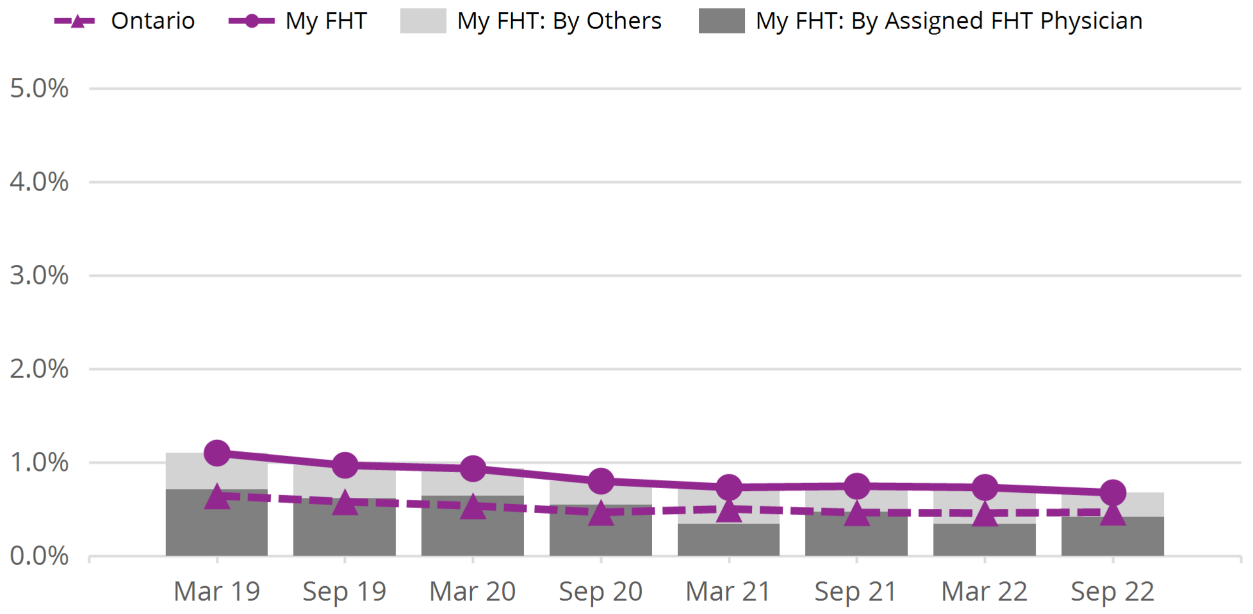

† Data suppressed where counts are between 1 and 5; additional suppression may be applied where counts are greater than 5 to prevent residual disclosure of suppressed values; N/A: Data not available; ¨ Please interpret with caution, denominator ≤ 30. For more details, refer to the Methods section on page 24.

Palliative care patients are not included; they were identified from hospital and physician billing claims data. For this indicator, the opioid medications' definition does not include opioid agonist therapy, opioid cough or anti-diarrheal medications.

**Number of my FHT's patients with a high-dose opioid >90mg MEQ daily within the last 6 months**
By their physician within the FHT: **61** By Other Providers within or outside of the FHT: **39**

Your patients who have pain need your primary care team.

Moderate quality evidence suggests a dose-dependent increase in risk as the prescribed dose of opioids increases. Some patients may gain important benefit at a dose of more than 90 mg MEQ daily (8). The data need to be interpreted in that context.

How many of my FHT's patients with chronic non-cancer pain are taking opioids outside of the recommended use guidelines? (page 13)

Ontario Health

Quality Improvement Ideas

How to Improve Opioid Prescribing in my Practice

Use the following steps to proactively deliver appropriate pain management and guide improvements for safe opioid prescribing in your practice.

Step 1: Identify patients prescribed an opioid for pain using your EMR

GENERATE a registry of patients prescribed an opioid for pain by using:

- ICD-10 diagnostic codes for pain (781, 847, 780, 346, 726, 787, 350)
- EMR keyword search for opioid medications

IDENTIFY the patients on this registry who have been prescribed an opioid:

- By you
- By other prescribers (data available through the Digital Health Drug Repository)

ASSESS the quality of your registry:

- Assess data quality and review your inclusion and exclusion criteria to ensure your registry is complete and correct
- Consider creating a separate list of patients in pain but not on opioids

Resources to help create an up-to-date registry:

EMR support

- Getting Started With an Opioid Use Registry (AFHTO)

In-person support

- OMD Advisory Service (OntarioMD)
- EMR Practice Enhancement Program (OntarioMD)
- Quality Improvement Decision Support Specialists (AFHTO)

To proactively deliver safe management, document every patient encounter completely and accurately in your EMR so that you can extract practice and patient level data electronically.

Step 2: Review current treatment plans for patients with pain to identify gaps in the provision of evidence based care

ASSESS the management plans of the patients on the registry to identify opportunities for improvement.

IDENTIFY which (and how many) patients meet the following criteria:

- Are at risk for opioid use disorder
- Are on a tapering regime or have had their opioid prescriptions abruptly stopped
- Have been prescribed opioids outside of the Canadian Guideline for Opioids for Chronic Non-Cancer Pain
- Are outside of the National Guideline for the Clinical Management of OUD (CRISM) for the use of opioid agonist treatment
- Are prescribed opioids for acute (<3 months) or chronic (>3 months) pain
- Have had a documented comprehensive assessment for pain (and assess how often this has been reviewed)
- Have a multimodal treatment plan to manage pain (e.g., self management, psychological supports, non-opioid pharmacological options)

Evidence-based guidelines and recommendations:

For opioid prescribing

- Opioid Prescribing for Acute Pain (Ontario Health)
- Opioid Prescribing for Chronic Pain (Ontario Health)
- Opioid Tapering Template (CEP)
- Opioid Tapering Template (RxFiles)
- Opioid Wisely Recommendations (Choosing Wisely)

For treating patients with opioid use disorder

- Opioid Use Disorder Quality Standard (Ontario Health)

Quality Improvement Ideas

How to Improve Opioid Prescribing in my Practice

STEP 3: Explore opportunities for improvement

The following are common actionable themes and resources that can help support your quality improvement initiatives in safer opioid prescribing.

Connect with peers and external supports

- Contact a practice facilitator to help build capacity for improvement
  - Quality improvement decision support specialists (AFHTO)
  - FHTs/NPLCs/CHCs: Resources are available on their Trello Board platform. Email improve@afhto.ca to sign up and gain access
- Participate in practice coaching and peer group mentorship
  - Peer Leaders (OntarioMD)

Access continuing education

- Explore continuing professional development opportunities:
- MacHealth's Opioids Clinical Primer (McMaster University)
- Collaborative Mentoring Networks (Ontario College of Family Physicians)
- Ontario Chronic Pain and Opioid Stewardship (Project ECHO)
- Safer Opioid Prescribing Program (University of Toronto)

Redesign your system and leverage digital health solutions

- Ensure your systems and processes support comprehensive assessments for people with pain
- Develop a virtual care strategy to support patient care management
  - Adopting and Integrating virtual visits into care (Ontario Health)
- Develop EMR reminder systems to recall patients due for tests, medication review, or examination
- Map your team's care process using QI Process Mapping Instructions
- Measure impact by using process indicators included in the opioid quality standards (Section: "How Success Can Be Measured") to guide your QI initiatives

Patient engagement

- Use the following patient reference guides to support conversations with your patients
  - Opioid Prescribing for Acute Pain
  - Opioid Prescribing for Chronic Pain
  - Opioid Use Disorder
- Consider using a treatment agreement to document informed consent and clarify expectations for both patient and physician
- Use Ontario Health's Patient Partnering Framework to guide planning, implementing, and evaluating patient partnering activities

Find more resources at Ontario Pain Management Resources: A partnership to help clinicians support their patients.

Join the conversation on Quorum:
1. Opioid Stories **2.** Opioids Indicators page **3.** Review Quorum's QI Tools & Resources for information and guidance on conducting quality improvement initiatives.

Ontario Health

What percentage of my eligible patients aged 50-74 are up-to-date with any colorectal screening?

- As of September 30, 2022, 73.7% of patients in my FHT were up-to-date with colorectal screening. My LHIN percentage is 66.2%.
- My FHT is **higher than** the provincial percentage of 62.5%.

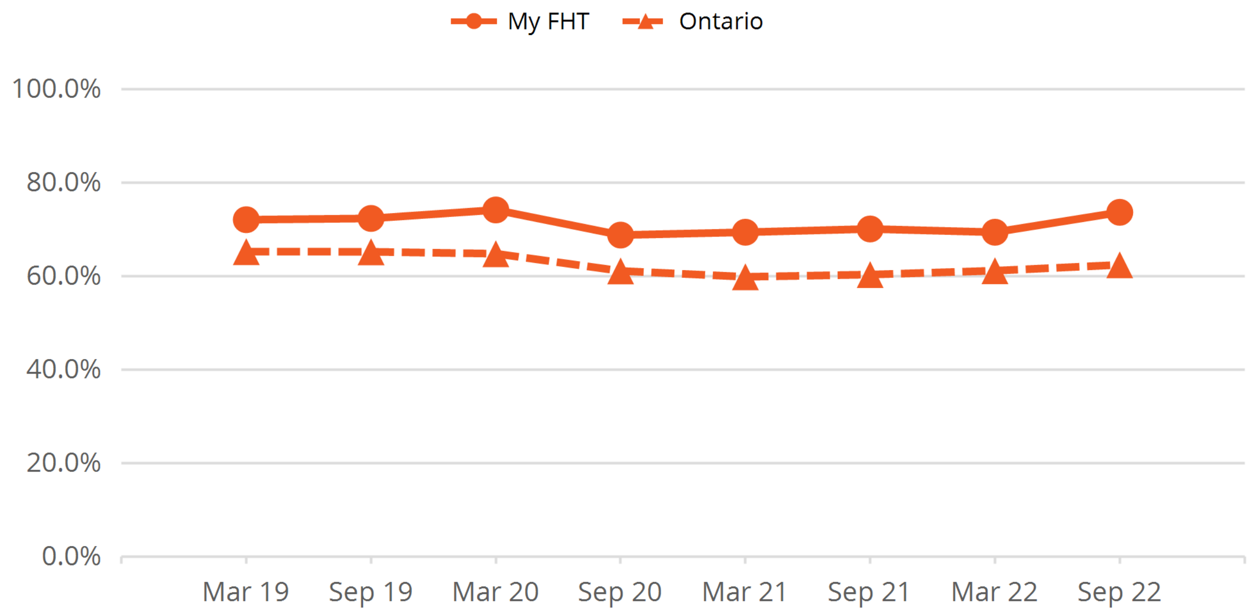

† Data suppressed where counts are between 1 and 5; additional suppression may be applied where counts are greater than 5 to prevent residual disclosure of suppressed values; N/A: Data not available; ¨ Please interpret with caution, denominator ≤ 30. For more details, refer to the Methods section on page 24.

Beginning with the September 2022 data cycle, the CRC screening indicator has been updated (new age range and inclusion of patients with inflammatory bowel disease). Please see the Technical Appendix for details.

A small proportion of FOBTs performed as diagnostic tests could not be excluded from the analysis.

**Number of my FHT's eligible patients not screened**
**1,434**

Evidence for screening continues to evolve. We will continue to monitor screening guidelines and modify the indicator, as appropriate (9).

Tests performed in hospital laboratories or paid through alternative payment plans are not captured.

How can my FHT improve its CRC screening? (page 18)

Identify your patients requiring follow up for cancer screening, through Ontario Health's screening activity report (SAR)

SAR Report Portal

Ontario Health

Pap Smear Screening

Data as of September 30, 2022

What percentage of my FHT's eligible patients aged 21-69 are up-to-date with Pap smear screening within the past three years?

- As of September 30, 2022, 65.2% of patients in my FHT had an up-to-date Pap smear test. My LHIN percentage is 54.7%.
- My FHT is **higher than** the provincial percentage of 50.6%.

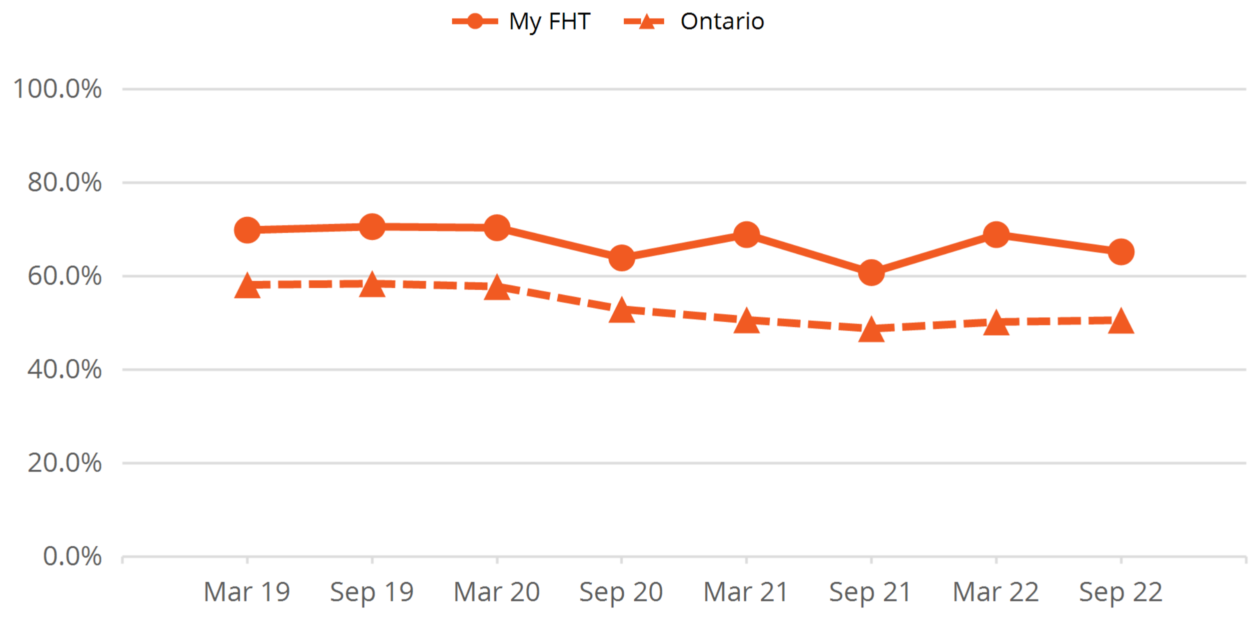

† Data suppressed where counts are between 1 and 5; additional suppression may be applied where counts are greater than 5 to prevent residual disclosure of suppressed values; N/A: Data not available; ¨ Please interpret with caution, denominator ≤ 30. For more details, refer to the Methods section on page 24.

Patients who have had cervical cancer, and patients who have had a hysterectomy are excluded.

Beginning with the September 2022 data cycle, the Pap smear screening indicator has been updated (new age range). Please see the Technical Appendix for details.

Number of my FHT's eligible patients not screened within the past three years
**1,393**

Evidence for cancer screening continues to evolve. We will continue to monitor screening guidelines and modify the indicator, as appropriate (10).

Tests performed in hospital laboratories or paid through alternative payment plans are not captured.

How can my FHT improve its Pap smear screening? (page 18)

Identify your patients requiring follow up for cancer screening, through Ontario Health's screening activity report (SAR)

SAR Report Portal

Ontario Health

Mammogram Screening

Data as of September 30, 2022

What percentage of my FHT's eligible patients aged 50-74 are up-to-date with mammogram screening within the past two years?

- As of September 30, 2022, 74.1% of patients in my FHT had an up-to-date mammogram. My LHIN percentage is 61.2%.
- My FHT is **higher than** the provincial percentage of 57.6%.

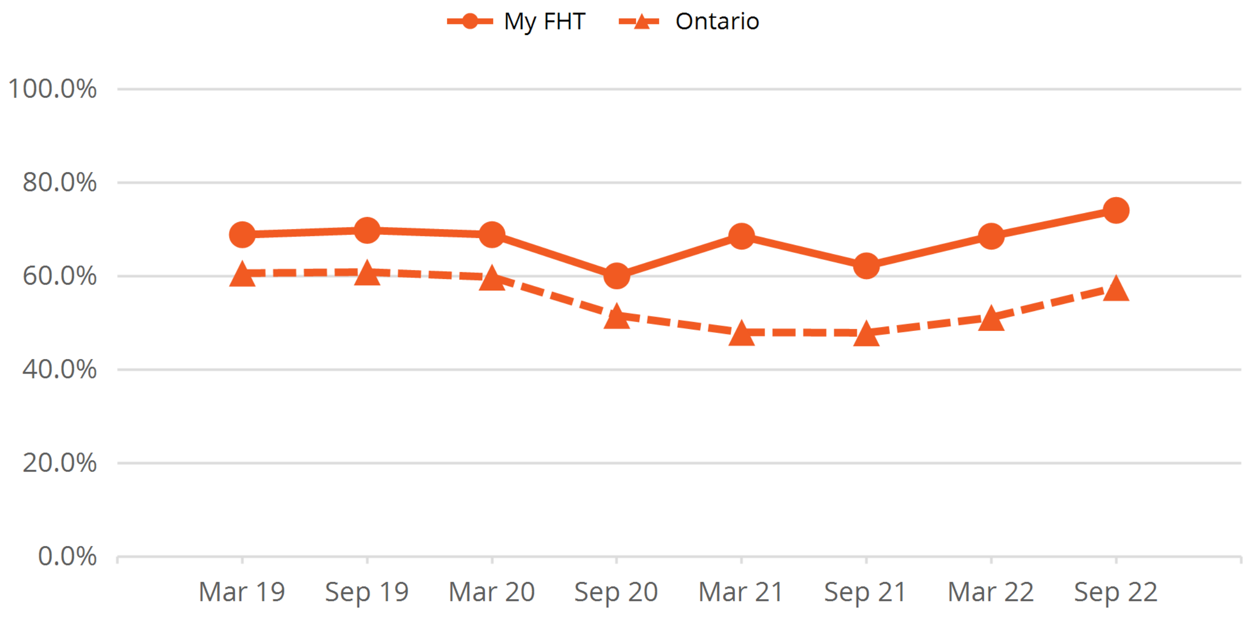

† Data suppressed where counts are between 1 and 5; additional suppression may be applied where counts are greater than 5 to prevent residual disclosure of suppressed values; N/A: Data not available; ¨ Please interpret with caution, denominator ≤ 30. For more details, refer to the Methods section on page 24.

Patients with a history of breast cancer are excluded.

Beginning with the September 2022 data cycle, the Mammogram screening indicator has been updated (new age range and exclusion of unilateral mammogram). Please see the Technical Appendix for details.

**Number of my FHT's eligible patients not screened within the past two years**
**705**

We recognize that the current recommendation is to have an active discussion with women about the benefits and limitations of breast screening (11-13). Some women who are eligible to be screened choose not to. Thus, the data need to be interpreted in that context.

How can my FHT improve its mammogram screening? (page 18)

Identify patients requiring follow up for cancer screening though Ontario Health's screening activity report (SAR)

SAR Report Portal

Ontario Health

Quality Improvement Ideas

How to Improve Cancer Screening in my Practice

Use the following steps to proactively guide improvements for cancer screening in your practice.

STEP 1: Identify patients eligible for cancer screening

Identify and verify which patients are eligible/due/overdue for cancer screening.

REGISTER for and view your Ontario Health Screening Activity Report (SAR) to view the screening status of your patients. You can appoint a delegate(s) from your primary care team to access your SAR.

GENERATE an up-to-date list of patients due/overdue for screening by running a report in your EMR and comparing that output with your Ontario Health SAR. Additional guidance:

- Optimally using your EMR for Cancer Screening - Hamilton Niagara Halton Brant Regional Cancer Program

REVIEW the accuracy of your data. Additional guidance:

- Useful Resources on Prevention and Screening - OntarioMD

REFLECT on understanding your patient population/community and consider your patients who have not been screened might be affected by issues of equity. Additional guidance:

- Poverty: A Clinical Tool for Primary Care - Centre for Effective Practice
- Success story - TAIBU Community Health Centre in Toronto

STEP 2: Review current screening guidelines to identify gaps in the provision of evidence-based care in screening

Make sure you review and are up to date on current guidelines and advice from Ontario Health - Cancer Care Ontario.

Breast Cancer Screening Guidelines	Cervical Screening Guidelines Summary	Colorectal Cancer Screening Recommendations Summary

MAP your practice's current cancer screening process by outlining the steps involved and the people responsible. This will help you identify the inefficiencies and opportunies for improvement. Ensure your systems and processes support comprehensive screening management, notification of results, and timely follow up. Additional Guidance:

- QI Process Mapping Instructions - Ontario Health

REFLECT on the efficiency and effectiveness of your process and set goals for your quality improvement initiatives. Use an up-to-date list of patients due/overdue for screening to set goals, including numerical, time-sensitive, equity-focused targets (how many patients are screened by which dates, while ensuring equity and access).

Quality Improvement Ideas

How to Improve Cancer Screening in my Practice

STEP 3: Explore opportunites for improvement

The following are common actionable themes and resources that can help support your quality improvement initiatives in cancer screening.

Redesign your system and leverage digital health solutions	Engage your patients	Access continuing education	Connect with peers and external supports
Design and test a virtual care strategy to support patient care management<br><br>• Clinical Guidance for Person-Centred Virtual Cancer Care - Ontario Health<br>• Adopting and Integrating virtual visits into care - Ontario Health<br><br>Develop EMR reminder systems to recall patients due for cancer screening.<br><br>• Tip: Consider identifying a champion on your team who can help or contact OntarioMD's Peer Leader Program for additional support	Update your process to track and engage patients eligible for screening<br><br>• Use these sample letters to create screening reminder letters for your patients, or sign up for physician linked correspondence for personalized letters that Ontario Health sends to remind patients to get screened<br>• Update EMR when reminder notices are issued. Regularly review list of patients due/overdue. Focus on engaging with those who experience inequity or marginilization in conversations about cancer screening<br>• Visit Ontario Health's Cancer Screening Resources Hub to access their provider toolkit and other resources.<br><br>Access reference guides to engage patients in conversations about their care<br><br>• Patient Involvement in decisions about care - Ontario Health's Quorum<br>• Patient Partnering Framework - Ontario Health	Explore continuing professional development opportunities to stay current on new guidelines and emerging cancer screening information<br><br>• Cancer 101 Toolkit for First Nations, Inuit and Métis People - Ontario Health<br><br>• COVID-19 Cancer Screening Tip Sheet for Primary Care Providers - Ontario Health<br><br>• Management of cancer screening services during the COVID-19 pandemic - Canadian Partnership Against Cancer<br><br>• Guidance for adenopathy related to vaccination - Ontario Health<br>• Screening Resources for Healthcare Providers - Ontario Health<br><br>• Contact Ontario Health for cancer screening continuing professional development presentations at primarycareinquiries@ontariohealth.ca<br><br>• Access Quorum's Quality Improvement QI Tools and Resources from Ontario Health to support each step in your journey	Work in collaboration with your Ontario Health Teams (OHT) teams to spread local changes together to impact care in your OHT region<br><br>Contact a practice facilitator to help build capacity for improvement<br>• Quality improvement decision support specialists - AFHTO<br>• FHTs/NPLCs/CHCs: Resources are available on their Trello Board platform. Email improve@afhto.ca to sign up and gain access<br><br>Participate in practice coaching and peer group mentorship<br>• Peer Leaders - OntarioMD<br>• OMD Advisory Service - OntarioMD<br>• EMR Practice Enhancement program - OntarioMD<br><br>Learn From your Peers<br>• Contact Ontario Health at primarycareinquiries@cancercare.on.ca to:<br>• Be connected to your local Regional Primary Care Lead and Regional Indigenous Care Lead<br>• Sign up to receive the Provincial Primary Care and Cancer Network monthly newsletter

Ontario Health

Diabetes Section | HbA1c Testing to Prevent Complications from Diabetes

Data as of September 30, 2022

What percentage of my FHT's patients with diabetes had two or more HbA1c tests within the past 12 months?

- As of September 30, 2022, 62.7% of patients with diabetes in my FHT were up-to-date with HbA1c testing. My LHIN percentage is 45.4%.
- My FHT is **higher than** the provincial percentage of 48.8%.

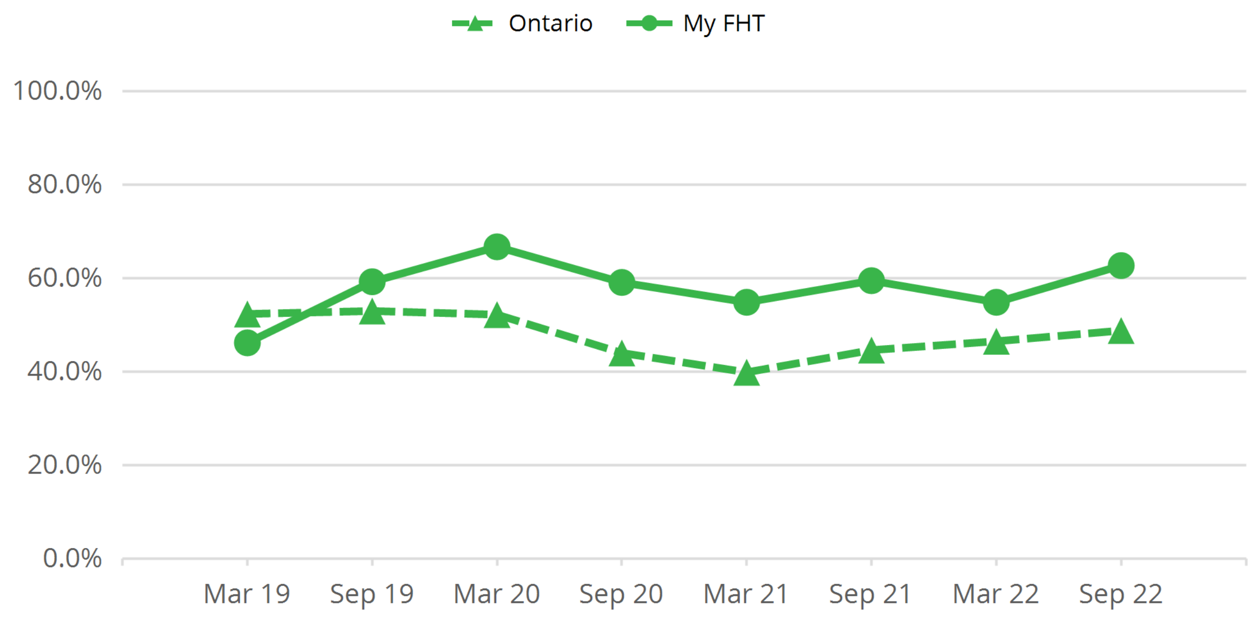

† Data suppressed where counts are between 1 and 5; additional suppression may be applied where counts are greater than 5 to prevent residual disclosure of suppressed values; N/A: Data not available; ¨ Please interpret with caution, denominator ≤ 30. For more details, refer to the Methods section on page 24.

This indicator does not differentiate between type I and type II diabetes but does exclude gestational diabetes.

**Number of my FHT's patients with diabetes with fewer than two HbA1c tests within the past 12 months**
**564**

Ontario Health will continue to monitor testing guidelines and adjust the indicator, as appropriate (14).

Tests performed in hospital laboratories or paid through alternative payment plans are not captured.

How can my FHT improve its HbA1c screening? (page 23)

Ontario Health

Retinal Exam Testing to Prevent Complications from Diabetic Retinopathy

Data as of September 30, 2022

What percentage of my FHT's patients with diabetes are up-to-date with retinal testing with an ophthalmologist or optometrist within the past 24 months?

- As of September 30, 2022, 70.0% of patients with diabetes in my FHT had an up-to-date retinal exam. My LHIN percentage is 62.1%.
- My FHT is **higher than** the provincial percentage of 63.6%.

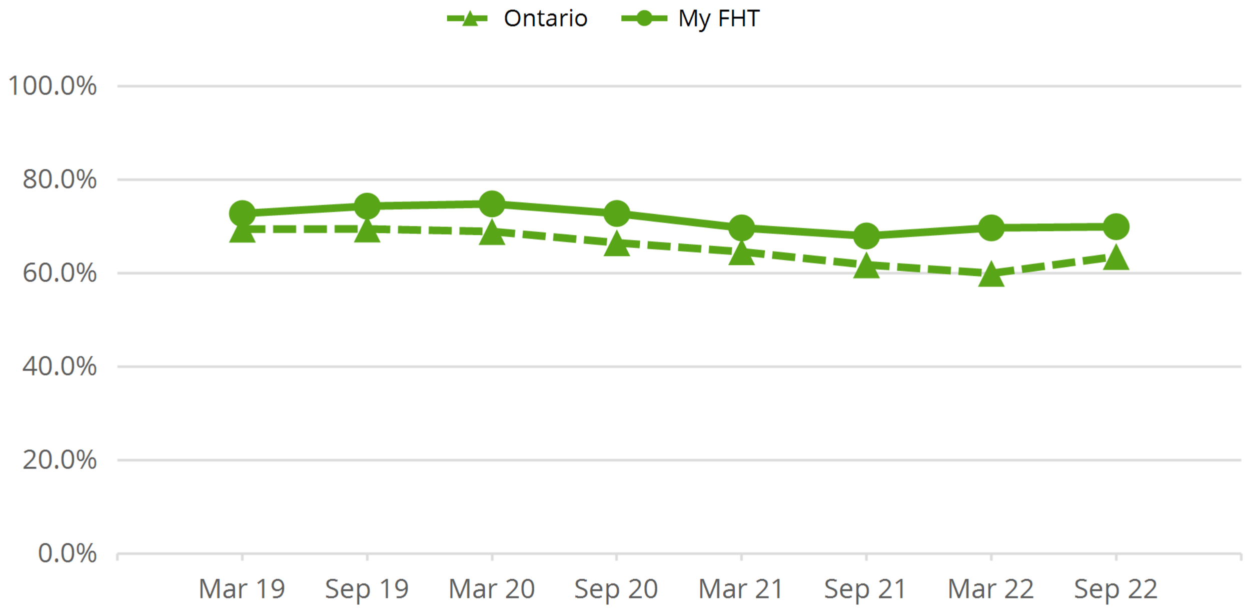

† Data suppressed where counts are between 1 and 5; additional suppression may be applied where counts are greater than 5 to prevent residual disclosure of suppressed values; N/A: Data not available; ¨ Please interpret with caution, denominator ≤ 30. For more details, refer to the Methods section on page 24.

This indicator does not differentiate between type I and type II diabetes but does exclude gestational diabetes.

Number of my FHT's patients with diabetes not tested within the past 24 months
454

Ontario Health will continue to monitor testing guidelines and adjust the indicator, as appropriate (15).

Tests performed in hospital laboratories or paid through alternative payment plans are not captured.

How can my FHT improve its retinal exam rate? (page 23)

Ontario Health

Statins Dispensed to Prevent Vascular Complications from Diabetes

Data as of September 30, 2022

What percentage of my FHT's patients with diabetes aged 66 and older have been dispensed a statin within the past 12 months?

- As of September 30, 2022, 78.9% of patients with diabetes in my FHT were dispensed a statin. My LHIN percentage is 74.0%.
- My FHT is **higher than** the provincial percentage of 74.2%.

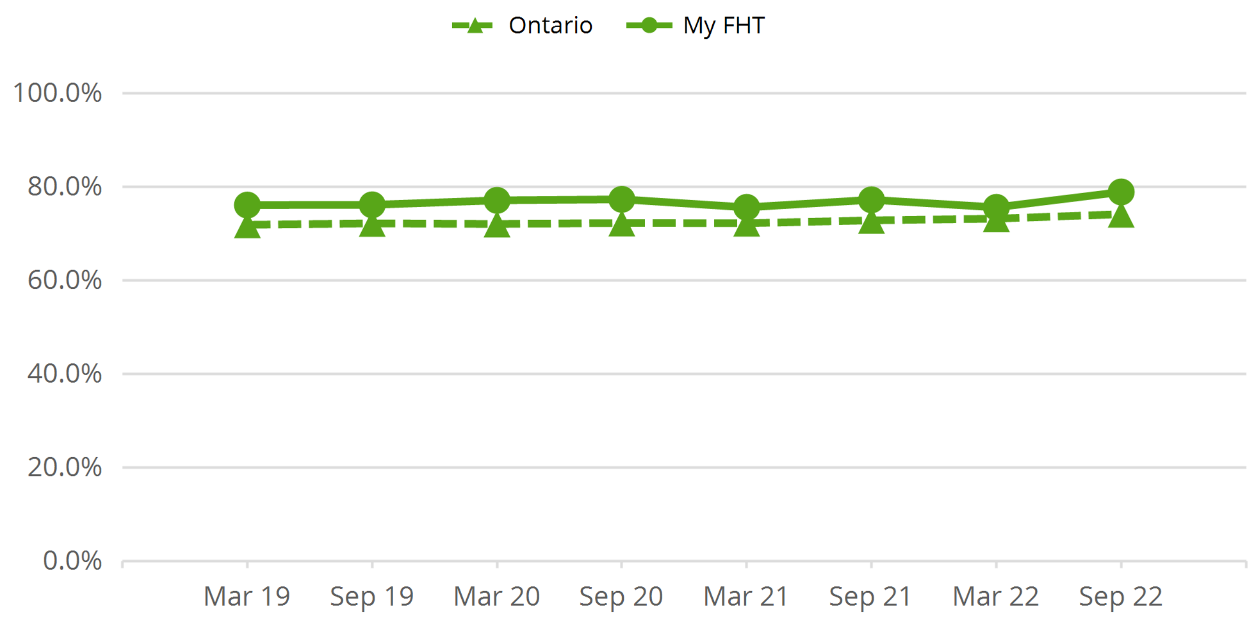

† Data suppressed where counts are between 1 and 5; additional suppression may be applied where counts are greater than 5 to prevent residual disclosure of suppressed values; N/A: Data not available; ¨ Please interpret with caution, denominator ≤ 30. For more details, refer to the Methods section on page 24.

This indicator does not differentiate between type I and type II diabetes. Data are not available for patients with diabetes below the age of 65 as they are not included in the ODB program. Prescriptions purchased outside of the ODB program are not included.

**Number of my FHT's patients with diabetes who were not dispensed a statin within the past 12 months**
**196**

Statin prescriptions may be more appropriate for some patients than others depending on baseline level of risk, co-morbid conditions, patient preferences and life expectancy (16-17).

How can my FHT improve its statin dispensing rate? (page 23)

Ontario Health

Quality Improvement Ideas — How to Improve Diabetes Management in my Practice

Use the following steps to help guide improvements for diabetes management in your practice

STEP 1: Identify people living with prediabetes and diabetes

- **GENERATE** an up-to-date registry of people with prediabetes by running an EMR search for ICD-10 diagnostic code 249 (prediabetes)
- **GENERATE** an up-to-date registry of people living with diabetes by running an EMR search for the following ICD-10 diagnostic codes:
  - 250 (diabetes mellitus)
  - 251 (other disorders of pancreatic internal secretions)
- **ASSESS** the quality of your registries/data:
  - Is my registry list complete?
  - Are there included and excluded patients that are incorrectly labelled?
  - Do I need to clean up my data prior to creating my registry to ensure correctness?

Resources to help create an up-to-date registry:

- EMR Practice Enhancement Program (OntarioMD)
- EMR Queries: Diabetes (AFHTO)
- OMD Advisory Service (OntarioMD)

To proactively deliver safe management, document every patient encounter completely and accurately in your EMR so that you can extract practice and patient level data electronically

STEP 2: Review current management plan of prediabetes and diabetes

- **REVIEW** your management of each patient living with **prediabetes, Type 1 diabetes, Type 2 diabetes**, and **diabetes in pregnancy** on the registry to ensure treatment plans are current with evidence-based management, and that their elements of care are assessed at regular intervals.
- **REFER** to the Ontario Health Quality Standards for guidance on how to deliver high-quality care
  - Type 1 Diabetes
  - Prediabetes and Type 2 Diabetes
  - Diabetes in Pregnancy
- **REFLECT** on the following questions to identify opportunities for improvement:
  - Am I using the correct diabetes care flow sheet?
  - Am I using the most up to date clinical practice guidelines?
  - Have management plans and lab results been reviewed in the last 3 months?
  - Is my documentation on elements of care up to date?

Resources for management support:

- Clinical Practice Guidelines (Diabetes Canada)
- Framingham Risk Score (Canadian Cardiovascular Society)
- Sample Diabetes Patient Care Flow Sheet for Adults (Diabetes Canada)

To efficiently and effectively document your management, consider uploading fillable forms (e.g., diabetes care flow sheet, Framingham Risk Score) to your EMR. These forms can be found in repositories of your EMR providers

Quality Improvement Ideas | How to Improve Diabetes Management in my Practice

STEP 3: Explore opportunities for improvement

The following are common actionable themes and resources that can help support your quality improvement initiatives in diabetes management.

Connect with peers and external supports

- Contact a practice facilitator to help build capacity for improvement
  - Quality improvement decision support specialists (AFHTO)
  - FHTs/NPLCs/CHCs: Resources are available on their Trello Board platform. Email improve@afhto.ca to sign up and gain access
- Consider referral pathways that improve patients outcomes such as Diabetes Education Programs, self management group, registered dieticians, etc.
- Participate in practice coaching and peer group mentorship
  - Peer Leaders (OntarioMD)

Access continuing education

- Explore continuing professional development opportunities:
  - Diabetes Canada Webinars
  - Access tools, resources, and academic detailing for Type 2 Diabetes and Managing Type 2 Diabetes During COVID-19. (Centre for Effective Practice)
- Review best practices in delivering high-quality care from the Ontario Health Quality Standards
  - Diabetes (Type 1)
  - Diabetes (Prediabetes and Type 2)
  - Diabetes in Pregnancy

Redesign your system and leverage digital health solutions

- Ensure your systems and processes support comprehensive diabetes management
- Support access and collaboration with inter-professional care teams to address comprehensive health needs
- Develop a virtual care strategy to support patient care management
  - Adopting and Integrating virtual visits into care (Ontario Health)
- Develop EMR reminder systems to recall patients due for tests, medication review, or examinations
- Map your team's care process using QI Process Mapping Instructions
- Measure impact by using process indicators included in the opioid quality standards (Section: "How Success Can Be Measured") to guide your QI initiatives

Partner with patients

- Encourage patients to be active in their management plans
- Access reference guides to engage patients in conversations about their care using the following resources:
  - Quorum's Patient Involvement in decisions about care
  - Newly Diagnosed (Diabetes Canada)
  - The following quality standards patient conversation guides:
    - Diabetes (Type 1)
    - Diabetes (Prediabetes and Type 2)
    - Diabetes in Pregnancy
- Use Ontario Health's Patient Partnering Framework to guide planning, implementing, and evaluating patient partnering activities

Join the conversation on Quorum:

- Review Quorum's QI Tools & Resources for information and guidance on conducting quality improvement initiatives.

Health Service Utilization

Data as of September 30, 2022

Indicator	Definition	My FHT (unadjusted)	My FHT (adjusted)	My LHIN	Ontario
**Total ED visits**	Rate of total hospital emergency department visits per 1,000 patients	**666.4**	450.1*	314.7*	349.5
**Urgent ED visits**	Rate of urgent hospital emergency department visits measured as CTAS level 1-3 per 1,000 patients	**388.3**	319.7*	235.7*	254.3
**Less Urgent ED visits**	Rate of less urgent hospital emergency department visits measured as CTAS level 4-5 per 1,000 of your patients	**162.8**	127.4*	74.1*	93.7
**Hospital Readmissions within 30 days**	Percentage of hospital readmissions within 30 days of discharge among your admitted patients	**6.5%**	6.3%*	5.5%*	5.6%
**Hospital Readmissions within 1 year**	Percentage of hospital readmissions within 1 year of discharge among your admitted patients	**17.9%**	16.5%*	15.0%*	15.7%
**SAMI Score**	The mean ACG weight of expected resource use in your practice *(definition updated to include additional fee codes as of Sept 2016)*	**1.0**	N/A	1.2	1.0
**Visits to Own Physician**	Percent of visits to own physician (continuity of care)	**80.6%**	N/A	67.7%	71.5%
**Visits to Own Group**	Percent of visits to own physician group (continuity of care)	**84.6%**	N/A	72.6%	76.9%

* risk adjustment takes into account differences among patient populations to allow for fairer comparisons between your practice and other comparators. The adjustment is made for age, sex, rurality, income, and co-morbidities.

† Data suppressed where counts are between 1 and 5; additional suppression may be applied where counts are greater than 5 to prevent residual disclosure of suppressed values; N/A: Data not available; ¨ Please interpret with caution, denominator ≤ 30. For more details, refer to the Methods section on page 24.

Health Service Utilization

Data as of September 30, 2022

Indicator	Definition	My FHT (unadjusted)	My FHT (adjusted)	My LHIN	Ontario
**ACSC Admissions - Total**	Rate of hospital admissions for one or more of the following conditions: asthma, CHF, COPD, and diabetes per 1,000 patients	**3.8**	3.0*	2.2*	2.7
**ACSC Admissions - Asthma**	Rate of hospital admissions for asthma per 1,000 patients	†	0.2*	0.3*	0.3
**ACSC Admissions - CHF**	Rate of hospital admissions for CHF per 1,000 patients	**1.3**	0.9*	0.9*	1.0
**ACSC Admissions - COPD**	Rate of hospital admissions for COPD per 1,000 patients	**1.6**	1.0*	0.6*	0.8
**ACSC Admissions - Diabetes**	Rate of hospital admissions for diabetes per 1,000 patients	**1.3**	0.8*	0.5*	0.6

* risk adjustment takes into account differences among patient populations to allow for fairer comparisons between your practice and other comparators. The adjustment is made for age, sex, rurality, income, and co-morbidities.

† Data suppressed where counts are between 1 and 5; additional suppression may be applied where counts are greater than 5 to prevent residual disclosure of suppressed values; N/A: Data not available; ¨ Please interpret with caution, denominator ≤ 30. For more details, refer to the Methods section on page 24.

Use the following steps to help guide improvements for less urgent emergency department visits in your practice

We recognize there are many factors associated with emergency department visits that are outside your control. In some areas of the province, emergency departments may play a role in providing timely access for less-urgent primary care. Below you will find some suggestions that can help you better understand your patients' emergency department visits.

1 Identify and verify the patients who are going to the ED

- Are there any patterns associated with ED use (e.g. day of week, time of day)?
- Consider how issues of equity may be contributing to this pattern.

Learn from your peers	• Learn from others on Quorum, Ontario Health's quality improvement online community.

"I looked at my data and found that the hospital readmission numbers started to rise when I decided to stop doing inpatient services. So that's one of the areas we are focusing on, to better work with the local hospital to know when admissions and discharges are happening so we can target patients for a 7-day post hospital visit."
– Dr. Ben Stobo, Athens District Family Health Team

My FHT's Patient Demographics and Disease Cohorts

Data as of September 30, 2022

Percentage of Your Patients By Age Cohort

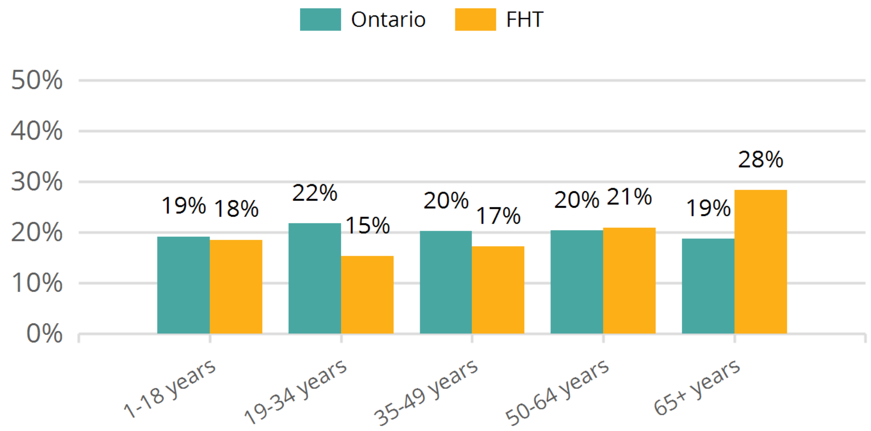

Percentage of Your Patients with Chronic Diseases

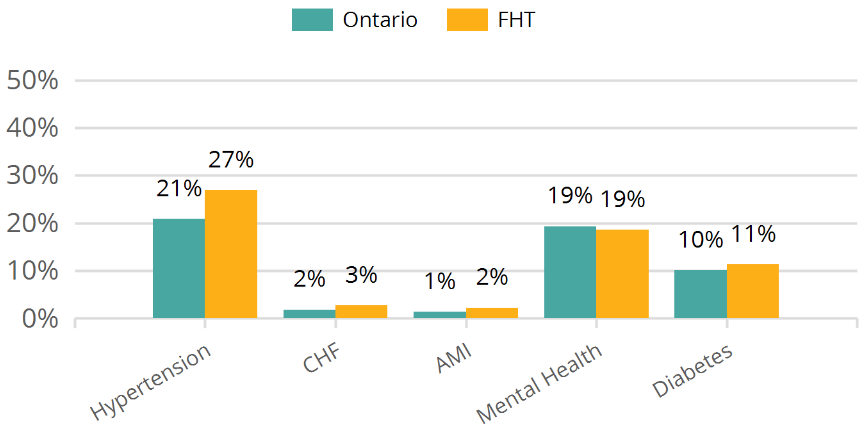

Percentage of Your Patients By Income Quintile

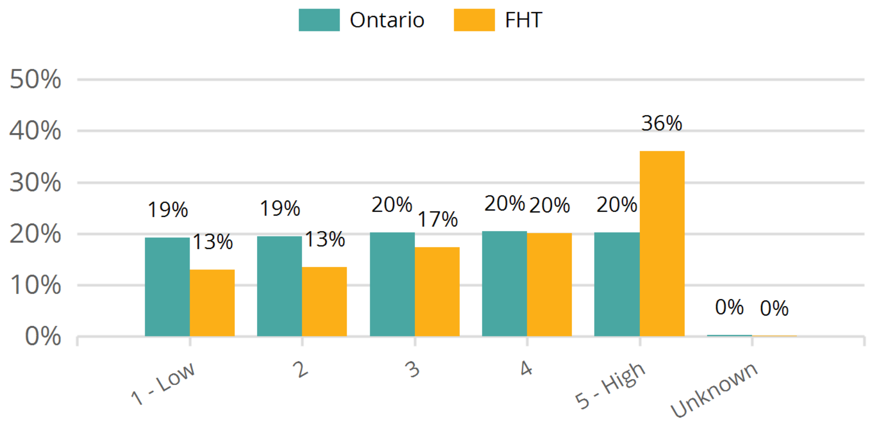

	My FHT	Ontario
Total Number of Patients	14,731	15,064,765
% Virtually Rostered	5.5%	9.5%
% Recent Immigrants	2.5%	12.5%
**Specialists Visits per 1,000 Patients**		
Cardiologist	54.9	86.5
Endocrinologist	49.1	56.7
Internal Medicine	95.4	97.9
Psychiatry	67.0	125.0
Respirologist	36.4	35.8

† Data suppressed where counts are between 1 and 5; additional suppression may be applied where counts are greater than 5 to prevent residual disclosure of suppressed values; N/A: Data not available; ¨ Please interpret with caution, denominator ≤ 30. For more details, refer to the Methods section on page 24.

"I was particularly interested to see the demographics data on my patients, especially the income quintiles. While our EMRs are able to provide us with lots of clinical data, a snapshot of the socioeconomic status of our practices is something completely unique to the MyPractice: Primary Care Reports."
- Dr. Mario Elia, Family Physician, London Ontario

Ontario Health

Methods and Acknowledgements

Your FHT physicians' College of Physicians and Surgeons of Ontario (CPSO) number were used to identify the patients they cared for and the associated data for calculating the indicators that appear in this report.

Identifying your patients - To identify the patients whom your FHT physicians' cared for, their CPSO number were linked to health care administrative databases housed at ICES. The report includes patients rostered to your FHT's physicians and patients for whom those physicians were the highest billing provider for a set of core primary care Ontario Health Insurance Plan (OHIP) fee codes. To find out more about this process, please refer to the Technical Appendix.

Indicator calculation - After your patients were identified, ICES used various administrative datasets to calculate each indicator. For instance, to calculate the percentage of patients with diabetes who had two or more glycated hemoglobin (HbA1C) tests within the past 12 months, OHIP claims and hospitalization records were used to identify patients with diabetes, and those who had HbA1C tests. The data sources and details about how each indicator is calculated can be found in the Technical Appendix.

Data sources - Administrative databases that were used to generate this report include: The Registered Persons Database (RPDB) for patient demographic information; the OHIP database for physician claims data; the National Ambulatory Care Reporting System (NACRS) database for emergency department visits; the Discharge Abstract Database (DAD) for hospitalization records, the Narcotics Monitoring System (NMS) for dispensing data. For a complete list of databases used, please refer to the Technical Appendix.

Data Interpretation Considerations:

Data suppression – Data are suppressed or additionally suppressed as per ICES' privacy policy for the following reasons: (a) Counts or summary statistics are between 1 and 5; or (b) To prevent residual disclosure of suppressed values.

Data comprehensiveness - Administrative databases cannot capture all the information that we would like when calculating these indicators. Limitations of the data to consider when reviewing this report are detailed in the table below.

What information is not included in the report?	What does this mean for me?
For diabetes management indicators, prescriptions for those under 65 years of age are not captured.	All diabetes management prescription related indicators only apply to your patients with diabetes who are older than 65.
Tests performed at hospital laboratories are not captured (e.g. HbA1c testing).	If your patients' tests are performed at a hospital laboratory, your test rates will appear **lower** than the actual rate.
Palliative care patients identified from hospital and physician billing claims data are excluded from all indicators.	There may be a slight difference in values for a number of indicators in this report compared to previous reports where palliative patients were included.

Methods and Acknowledgements (continued)

Data timeliness - Data included in this report are not as current as would be preferred. However, they do provide a snapshot of your performance at a moment in time and a comparison to your peers at the group (if you are in a PEM), LHIN and Ontario levels for context. While HQO and our partners are always looking for ways to provide more timely data, we encourage you to also use local data sources to track and measure your progress.

Calculations used to generate indicators are not perfect - Complex calculations are used to translate information contained in the administrative databases into useful indicators. These calculations have been validated and/or used by researchers, public reporting or quality improvement initiatives. However, they are not always perfect. For instance, the number of diabetes patients in this report is unlikely to be a perfect match to the number identified through your EMR. In spite of this, information provided in this report provides a starting point so that you can assess your performance with your peers.

About Ontario Health

For information about Ontario Health, please visit www.ontariohealth.ca.

About ICES:

This study was supported by ICES, which is funded by an annual grant from the Ontario Ministry of Health and Long-Term Care (MOHLTC). The opinions, results and conclusions reported in this paper are those of the authors and are independent from the funding sources. No endorsement by ICES or the Ontario MOHLTC is intended or should be inferred. Parts of this material are based on data and information compiled and provided by CIHI. However, the analyses, conclusions, opinions and statements expressed herein are those of the author, and not necessarily those of CIHI.

Acknowledgements:

Many individuals contributed to the realization of this report, both internal and external to Ontario Health and ICES. We would like to acknowledge Dr. Rick Glazier and colleagues at ICES; Dr. Noah Ivers and colleagues at Women's College Hospital; Dr. Kevin Schwartz and Bradley Langford at Public Health Ontario; Dr. Jerome Leis, Sunnybrook Research Institute; and Drs. Anne Fraser and Anthony Train, family physicians. We are grateful for the input and support of the Association of Family Health Teams of Ontario (AFHTO) and the Ontario College of Family Physicians (OCFP) and to the clinicians, executive directors and decision support/quality improvement specialists who participated in our working group meetings during which valuable insight was provided on the report from a user's perspective.

Participation and confidentiality:

You are receiving this report because you have registered at Ontario Health's website. Your MyPractice report will only be sent to the validated email address you provided upon registration and will not be shared with others, including other agencies, the college, physician groups, or other members of your team.

Methods and Acknowledgements (continued)

The recipient and/or viewer of these reports is not permitted to use the aggregate and/or de-identified information in the reports, either alone or with other information, to identify an individual. This includes attempting to decrypt information that is encrypted, attempting to identify an individual based on unencrypted information, and attempting to identify an individual based on prior knowledge.

References

1. Public Health Agency of Canada. (2020). Antimicrobial resistance surveillance system report. https://www.canada.ca/content/dam/hc-sc/documents/services/drugs-health-products/canadian-antimicrobial-resistance-surveillance-system-2020-report/CARSS-2020-report-2020-eng.pdf (accessed October 8, 2021)
2. Schwartz, K.L., Langford, B.J., Daneman, N., Chen, B., Brown, K.A., McIsaac, W., et al. (2020). Unnecessary antibiotic prescribing in a Canadian primary care setting: a descriptive analysis using routinely collected electronic medical record data. CMAJ Open 8(2): E360-E369.
3. Silverman, M., Povitz, M., Sontrop, J.M., Li, L., Richard, L., Cajic, S., Shariff, S.Z. (2017). Antibiotic prescribing for nonbacterial acute upper respiratory infections in elderly people. Annals of Internal Medicine 166(11): 765-774.
4. Dawson-Hahn et al. (2017). Short-course vs. long-course oral antibiotics for infections treated in outpatient settings: a review of systematic reviews. Family Practice 34(5): 511-519.
5. Milo G, Katchman EA, Paul M, Christiaens T, Baerheim A, Leibovici L. Duration of antibacterial treatment for uncomplicated urinary tract infection in women. (2005). Cochrane Database Syst Rev. 2005;(2):CD004682.
6. Vaughn, V.M., Flanders, S.A., Snyder, A., Conlon, A., Roger, M.A.M., Malani, A.N., et al. (2019). Excess antibiotic treatment duration and adverse events in patients hospitalized with pneumonia: a multihospital cohort study. Annals of Internal Medicine 171(3): 153-163.
7. Grant, J., Le Saux, N., members of the Antimicrobial Stewardship and Resistance Committee (ASRC) of the Association of Medical Microbiology and Infectious Disease (AMMI). (2021). Duration of antibiotic therapy for common infections. JAMMI 6(3): 181-197.
8. Busse, J. W., Craigie, S., Juurlink, D. N., Buckley, D. N., Wang, L., Couban, R. J., . . . Guyatt, G. H. (2017). Guideline for opioid therapy and chronic noncancer pain. Canadian Medical Association Journal, 189(18), E659-E666.
9. Tinmouth, J., Vella, E., Baxter, N.N., Dubé, C., Gould, M., Hey, A., et al. (2015). Colorectal cancer screening in average risk populations: Evidence summary. Toronto (ON): CCO; November 11. Program in Evidence-based Care Evidence Summary No.: 15-14.
10. The Canadian Task Force on Preventive Health Care. (2013). Recommendations on screening for cervical cancer. Canadian Medical Association Journal, 185(1), 35-45.
11. Cancer Care Ontario. (2015, October). Information for Healthcare Providers on the Ontario Breast Screening Program (OBSP). Retrieved from https://www.cancercareontario.ca/sites/ccocancercare/files/assets/OBSPHealthcareProviders.pdf
12. Cancer Care Ontario. (n.d.). Screening for Breast Cancer. Retrieved February 25, 2019, from https://www.cancercareontario.ca/en/types-of-cancer/breast-cancer/screening

References

13. Klarenbach, S., Sims-Jones, N., Lewin, G., Singh, H., Thériault, G., Tonelli, M., ... & Thombs, B. D. (2018). Recommendations on screening for breast cancer in women aged 40–74 years who are not at increased risk for breast cancer. Canadian Medical Association Journal, 190 (49), E1441-E1451.
14. Berard, L. D., Siemens, R., Woo, V., & Diabetes Canada Clinical Practice Guidelines Expert Committee. (2018). Monitoring Glycemic Control. Canadian Journal of Diabetes, 42, S47-S53.
15. Altomare, F., Kherani, A., Lovshin, J., & Diabetes Canada Clinical Practice Guidelines Expert Committee. (2018). Retinopathy. Canadian Journal of Diabetes, 42, S210-S216.
16. Stone, J. A., Houlden, R. L., Lin, P., Udell, J. A., Verma, S., & Diabetes Canada Clinical Practice Guidelines Expert Committee. (2018). Cardiovascular protection in people with diabetes. Canadian Journal of Diabetes, 42, S162-S169.
17. Kutner, J. S., Blatchford, P. J., Taylor, D. H. Jr., Ritchie, C. S., Bull, J. H., Fairclouch, D. L., . . . Abernethy, A. P. (2015). Safety and benefit of discontinuing statin therapy in the setting of advanced, life-limiting illness: a randomized clinical trial. JAMA Internal Medicine, 175(5), 691 -700.
