# Supplementary material for: The engagement equation: a model for understanding what drives voluntary physician engagement with data-driven clinical performance feedback
Source: Implement Sci Commun. 2025 Dec 11;7:8. doi: 10.1186/s43058-025-00819-5 (PMC12801449; doi:10.1186/s43058-025-00819-5)
Supplement: Supplementary file 2 — Additional file 2. [file 43058_2025_819_MOESM2_ESM.docx]

**Appendix 2 - Confirmatory Factor Analysis**

|  | Robust CFI | Robust TLI | Robust RMSEA | SRMR | Scaled 𝛘^2^ | Scaled df |
| --- | --- | --- | --- | --- | --- | --- |
| 5 factors (original model) | 0.96 | 0.95 | 0.04 | 0.05 | 302.66 | 220 |
| 4 factors (*change discrepancy* and *feedback value* combined) | 0.88 | 0.86 | 0.07 | 0.08 | 440.32 | 224 |
| 3 factors (*change discrepancy* and *feedback value* combined, *feedback utility* and *feedback accountability* combined) | 0.87 | 0.85 | 0.07 | 0.08 | 459.12 | 227 |
| 2 factors (*change discrepancy* and *feedback value* combined, *feedback self-efficacy*, *feedback* *utility* and *feedback* *accountability* combined) | 0.74 | 0.71 | 0.11 | 0.10 | 685.08 | 229 |
| 1 factor | 0.66 | 0.62 | 0.12 | 0.10 | 807.41 | 230 |

N.B. CFI: Comparative Fit Index; TLI: Tucker–Lewis Index; RMSEA: Root Mean Square Error of Approximation; SRMR: Standardized Root Mean squared Residual
