# Supplementary material for: The engagement equation: a model for understanding what drives voluntary physician engagement with data-driven clinical performance feedback
Source: Implement Sci Commun. 2025 Dec 11;7:8. doi: 10.1186/s43058-025-00819-5 (PMC12801449; doi:10.1186/s43058-025-00819-5)
Supplement: Supplementary file 3 — Additional file 3. [file 43058_2025_819_MOESM3_ESM.docx]

Additional File 3

Construct Reliability and Heterotrait-Monotrait Ratio of Correlations

|  | Cronbach’s α | ρ_c_ | AVE | C1 | C2 | C3 | C4 | C5 |
| --- | --- | --- | --- | --- | --- | --- | --- | --- |
| Change discrepancy (C1) | 0.86 | 0.91 | 0.71 | -- |  |  |  |  |
| Feedback value (C2) | 0.78 | 0.86 | 0.61 | 0.59 | -- |  |  |  |
| Feedback self-efficacy (C3) | 0.86 | 0.90 | 0.64 | 0.22 | 0.43 | -- |  |  |
| Feedback utility (C4) | 0.82 | 0.87 | 0.58 | 0.58 | 0.78 | 0.60 | -- |  |
| Feedback accountability (C5) | 0.72 | 0.83 | 0.55 | 0.47 | 0.72 | 0.47 | 0.85 | -- |

N.B: AVE: Average Variance Extracted
