# Supplementary material for: The engagement equation: a model for understanding what drives voluntary physician engagement with data-driven clinical performance feedback
Source: Implement Sci Commun. 2025 Dec 11;7:8. doi: 10.1186/s43058-025-00819-5 (PMC12801449; doi:10.1186/s43058-025-00819-5)
Supplement: Supplementary file 4 — Additional file 4. [file 43058_2025_819_MOESM4_ESM.docx]

**Appendix 3 *-* Model Fit Indices for The Latent Profile Analysis**

| Model | No. of classes | AIC | BIC | SABIC | BLRT(p) | Entropy | Percentage of smallest group |
| --- | --- | --- | --- | --- | --- | --- | --- |
| 1 | 1 | 1762.80 | 1782.77 | 1763.76 | -- | 1.00 | 1.00 |
| 1 | 2 | 1579.15 | 1612.43 | 1580.74 | 0.010 | 0.81 | 0.27 |
| 1 | 3 | 1425.62 | 1472.21 | 1427.85 | 0.010 | 0.89 | 0.20 |
| 1 | 4 | 1364.20 | 1424.10 | 1367.07 | 0.010 | 0.86 | 0.09 |
| 1 | 5 | 1329.65 | 1402.86 | 1333.16 | 0.010 | 0.89 | 0.04 |
| 1 | 6 | 1319.92 | 1406.45 | 1324.07 | 0.020 | 0.88 | 0.03 |
| 2 | 1 | 1762.80 | 1782.77 | 1763.76 | -- | 1.00 | 1.00 |
| 2 | 2 | 1565.12 | 1608.38 | 1567.20 | 0.010 | 0.94 | 0.18 |
| 2 | 3 | 1399.34 | 1465.90 | 1402.53 | 0.010 | 0.86 | 0.22 |
| 2 | 4 | 1347.81 | 1437.67 | 1352.12 | 0.010 | 0.87 | 0.21 |
| 2 | 5 | 1291.33 | 1404.48 | 1296.75 | 0.010 | 0.89 | 0.10 |
| 2 | 6 | 1260.42 | 1396.86 | 1266.96 | 0.010 | 0.88 | 0.12 |
| 3 | 1 | 1193.67 | 1223.62 | 1195.10 | -- | 1.00 | 1.00 |
| 3 | 2 | 1198.16 | 1241.43 | 1200.24 | 0.525 | 0.59 | 0.16 |
| 3 | 3 | 1161.82 | 1218.39 | 1164.53 | 0.010 | 0.86 | 0.15 |
| 3 | 4 | 1169.46 | 1239.35 | 1172.81 | 0.822 | 0.60 | 0.01 |
| 3 | 5 | 1161.00 | 1244.19 | 1164.98 | 0.030 | 0.61 | 0.00 |
| 3 | 6 | 1168.58 | 1265.09 | 1173.20 | 0.792 | 0.65 | 0.04 |
| 6 | 1 | 1193.67 | 1223.62 | 1195.10 | -- | 1.00 | 1.00 |
| 6 | 2 | 1161.41 | 1224.64 | 1164.44 | -- | 0.49 | 0.35 |
| 6 | 3 | 1115.08 | 1211.59 | 1119.70 | -- | 0.65 | 0.27 |
| 6 | 4 | 1105.90 | 1235.69 | 1112.12 | -- | 0.72 | 0.17 |
| 6 | 5 | 1084.02 | 1247.09 | 1091.83 | -- | 0.74 | 0.15 |
| 6 | 6 | -- | -- | -- | -- | -- | -- |

N.B. Model 1: equal variances and covariances fixed to 0, Model 2: varying variances and covariances fixed to 0, Model 3: equal variances and equal covariances, and Model 6: varying variances and varying covariances. AIC: Akaike Information Criteria; BIC: Bayesian Information Criteria; SABIC: Sample Size–Adjusted BIC; BLRT: bootstrapped log-likelihood ratio tests.
