# Supplementary material for: The engagement equation: a model for understanding what drives voluntary physician engagement with data-driven clinical performance feedback
Source: Implement Sci Commun. 2025 Dec 11;7:8. doi: 10.1186/s43058-025-00819-5 (PMC12801449; doi:10.1186/s43058-025-00819-5)
Supplement: Supplementary file 5 — Additional file 5. [file 43058_2025_819_MOESM5_ESM.docx]

**Phase II: Semi-Structured Interviews**

**Research Questions:**

1. **How do primary care physicians think about practice feedback and improvement?**
2. **What distinguishes engagers from non-engagers?**
3. **What skills are needed to effectively engage?**

Semi-Structured Interview Guide (for participants who have accessed their MPPC Report)

The purpose of this interview is to explore your perspectives, beliefs, and experiences around Ontario Health’s My Practice: Primary Care report, as well as the factors that influence your ability to use this report to drive improvement in your practice and for your patients.

If you don’t have your report in front of you, please take a moment to pull it up before we begin.

I’d like to start the conversation by talking about what factors influenced your ability or desire to sign up to receive the report in the first place.

1. What motivated you to register for the MPPC report?
   1. TDF Social Influences
2. What role does feedback play for primary care physicians and the care they provide?
   1. Follow up: What kinds of things do you feel accountable for when it comes to feedback on your performance? *(FOS - Feedback Accountability)*

*Prompts: Who provides it, about what, underlying emotions*

1. How does feedback contribute to your success (or not) as a primary care physician? *(FOS Feedback Utility)*
2. How confident are you in your ability to act on the feedback you receive? *(FOS Feedback Self-Efficacy)*
   1. What factors contribute to your perceived confidence?
3. What is your overarching goal in your role as a physician? *(Regulatory Focus; TDF Professional Role & Identity)*
4. What current goals do you have related to quality of care in your practice and how do you ensure you achieve them? *(TDF Goals)*
   1. Follow up: How does that align, or not, with the feedback you receive on your performance?
5. How does the report benefit you or your patients? *(OCRBS Valence)*
6. There’s a theory around goal attainment that distinguishes between pursuing goals that promote gain versus those that avoid or prevent loss. What is your experience of how promoting gain versus avoiding loss plays out in your job? *(Regulatory Focus)*

Thank you so much, these insights are great. I’d like to shift our conversation to your use of or engagement with the report itself.

1. Can you walk me through what the report is telling you and what it means? *(TDF Skills)*
   1. Follow up: What information is most important to you in your report?
   2. Follow up: What do you see as the primary goal of this report?
2. How can practice-level data help inform your clinical decision-making? *(TDF Skills, FOS Feedback Utility)*
3. What matters most to you in your role as a physician? *(TDF Professional Role & Identity)*
   1. Follow up: How does that align, or not, with the data you receive in the report?
4. To what extent is it part of your job to understand your performance and identify areas for improvement? *(TDF Professional Role & Identity)*

We know that context, how feedback is designed, and the characteristics of the person receiving feedback influence whether or not it has a positive impact on care.

1. What report-level factors influence your ability to engage with performance feedback? *(CFIR – Characteristics of the Intervention)*
   1. Follow up: What does the report do well? What is missing from this report that would help drive performance improvement?
2. What contextual factors influence your ability to engage with performance feedback? *(CFIR – Outer setting)*
   1. Follow up: What impacts the care you provide that isn’t captured in the report?
   2. Follow up: How does your team use feedback (or other data) to improve your practice?
3. What physician-specific factors influence your ability to engage with performance feedback? *(CFIR – Characteristics of Individuals)*
4. What influences your ability to implement practice improvements as planned? *(CFIR – Process)*

*Confirm whether participant is interested in receiving their honorarium or information about the results of this study. If so, confirm email address*

Semi-Structured Interview Guide (for participants who have never accessed their MPPC Report)

The purpose of this interview is to explore your perspectives, beliefs, and experiences around using data to understand performance and inform practice improvement.

1. What role does feedback play for primary care physicians and the care they provide?
   1. Follow up: What kinds of things do you feel accountable for when it comes to feedback on your performance? *(FOS - Feedback Accountability)*

*Prompts: Who provides it, about what, underlying emotions*

1. How does receiving feedback contribute to your success (or not) as a primary care physician? *(FOS Feedback Utility)*
   1. Follow up: What makes feedback useful for you?
2. How confident are you in your ability to act on the feedback you receive? *(FOS Feedback Self-Efficacy)*
   1. What contributes to that?
3. What is your overarching goal in your role as a physician? *(Regulatory Focus; TDF Professional Role & Identity)*
4. What current goals do you have related to quality of care in your practice and how do you ensure you achieve them? *(TDF Goals)*
   1. Follow up: How does that align, or not, with the feedback you receive on your performance?
5. How do you know if you’re performing well against your goals?
   1. Follow up: How do you identify areas for improvement?
6. There’s a theory around goal attainment that distinguishes between pursuing goals that promote gain versus those that avoid or prevent loss. What is your experience of how promoting gain versus avoiding loss plays out in your job? *(Regulatory Focus)*
7. What are your thoughts on the value of implementing supports for primary care physicians to assist with improving quality of care? (*CFIR – Characteristics of Individual)*
   1. Follow up: What do you think drives physicians to engage with performance feedback?

Prompt: Is it to mitigate harm (prevention-focus)? Improve performance (promotion-focus)?

1. Can you give me examples of the type of thing(s) that influence the way you practice?
   1. Follow up: What type of thing(s) would lead you to make a change?
2. What influences your ability to achieve your performance goals or improvements as planned? *(CFIR – Process)*
3. What is your perspective on the value of using practice-level data to inform your clinical decision-making? *(TDF Skills)*
   1. Prompt: Can you walk me through what type of practice data might be useful and how it would help you?
4. What, if anything, would motivate you to engage with performance feedback? *(CFIR – Characteristics of the Intervention)*
5. What skills or supports would primary care physicians need to act on practice feedback data? *(TDF Skills)*
